# Muscle energy technique to reduce pain and disability in cases of non-specific neck pain: A systematic review and meta-analysis of randomized controlled trials

**DOI:** 10.1016/j.heliyon.2023.e22469

**Published:** 2023-11-17

**Authors:** Long-Huei Lin, Ting-Yu Lin, Ke-Vin Chang, Wei-Ting Wu, Levent Özçakar

**Affiliations:** a Kaohsiung Rukang Physiotherapy Clinic, Kaohsiung, Taiwan; b Department of Physical Medicine and Rehabilitation, Lo-Hsu Medical Foundation, Inc., Lotung Poh-Ai Hospital, Yilan, Taiwan; c Department of Physical Medicine and Rehabilitation, National Taiwan University Hospital, College of Medicine, National Taiwan University, Taipei, Taiwan; d Department of Physical Medicine and Rehabilitation, National Taiwan University Hospital, Bei-Hu Branch, Taipei, Taiwan; e Center for Regional Anesthesia and Pain Medicine, Wang-Fang Hospital, Taipei Medical University, Taipei, Taiwan; f Department of Physical and Rehabilitation Medicine, Hacettepe University Medical School, Ankara, Turkey

**Keywords:** Neck pain, Rehabilitation, Manual therapy, Physical therapy

## Abstract

**Background:**

To investigate the effectiveness of muscle energy technique (MET) for treatment of non-specific neck pain (NSNP).

**Methods:**

A literature search was performed using electronic databases from their inception until October 2023 for randomized controlled trials (RCTs) that investigated the effects of MET on NSNP. A change in pain intensity and reduced disability were the primary and secondary outcomes, respectively, standardized using Hedges’ *g*. A random effects model was used for data pooling.

**Results:**

This study included 26 RCTs comprising 1170 participants. The results showed that MET significantly reduced pain intensity (Hedges' *g* = −0.967 95 % CI = −1.417 to −0.517, *p* < 0.001). However, subgroup analysis revealed that this significant benefit was observed only when MET was combined with other treatments and not with MET monotherapy. MET also reduced disability (Hedges’ *g* = −0.545, 95 % CI = −1.015 to − 0.076, *p* = 0.023). Meta-regression analysis showed that an increase in treatment duration/session per week contributed to greater pain reduction. No adverse events were reported following the MET.

**Conclusions:**

In conclusion, our meta-analysis suggests MET's potential effectiveness within a combined treatment for NSNP. However, the evidence's low certainty is likely influenced by bias and study variations. To strengthen these findings, future research should focus on higher-quality clinical trials, longer follow-up periods, and prediction interval presentations.

## 1 Introduction

Neck pain is among the most frequently reported musculoskeletal disorders encountered in clinical practice, with a mean lifetime prevalence of 50.0 % [1]. It typically develops in the posterior cervical region and extends from the superior nuchal line to the scapular spine, superior border of the clavicle, and the suprasternal notch [2]. Similar to the difficulty encountered in patients with low back pain, it is challenging to identify the exact cause of neck pain in most patients, and the underlying pathology remains undetermined in 50.0–80.0 % of cases of back or neck pain [3]. Nonspecific neck pain (NSNP) is defined as neck pain without any known structural or neurological factors to account for the pain [4] and is commonly caused by postural and mechanical factors [5]. NSNP is also associated with maintaining a sustained neck posture or myofascial trigger points (MTrPs) in the neck [6].

The muscle energy technique (MET) is a form of manual therapy frequently used by physical therapists to improve musculoskeletal function and alleviate pain. MET includes stretching maneuvers after active muscle contraction and relaxation to improve joint mobility and restore normal muscle length. The two major MET techniques include post-isometric relaxation (PIR) and reciprocal inhibition (RI). PIR involves the application of a passive stretch to the muscle group, followed by isometric contraction of the same muscle group [7]. In contrast, RI involves contractions of the antagonist muscle group, which results in inhibition of the agonist muscle group [7]. PIR triggers the Golgi tendon reflex after contraction of the agonist muscle [8]. In contrast, RI results in muscle spindle activation secondary to stretching and produces reflexive contractions of the contracted muscle group. The reflexive contraction also produces relaxation of the antagonist muscle group, which promotes RI [9]. Several randomized controlled trials (RCTs) have reported MET as a promising therapeutic approach in cases of spinal pain, which has also been reported by many reviews [10,11]. A systematic review of 18 RCTs and three non-RCTs performed by Sbardella et al. [10] showed that MET may reduce neck pain and improve cervical range of motion. In a meta-analysis of 19 clinical trials, Santos et al. [11] reported that MET may have a moderate effect on pain reduction for subacute nonspecific low back pain; however, its benefit in reduction of disability was insignificant. However, no meta-analysis has specifically reported the effectiveness of MET in relieving pain and disability in patients with NSNP. We performed this meta-analysis to gain deeper insight into this subject. We hypothesized that, with the statistical power afforded by multiple studies, the MET would show pain reduction and disability improvement in the population with NSNP.

## 2 Methods

### 2.1 Search strategy

We performed a comprehensive search based on the Preferred Reporting Items for Systematic Reviews and Meta-Analyses (PRISMA) 2020 guideline [12]. Table S1 summarizes the PRISMA checklist. The protocol was registered on Inplasy.com under the registration number of INPLASY202340104. Two reviewers (L.-H.L. & T.-Y.L.) screened PubMed, ClinicalTrials.gov, Cochrane Library, Physiotherapy Evidence Datebase (PEDro) and Embase using the following keywords: “muscle energy technique” OR “post-isometric relaxation” OR “reciprocal inhibition” AND “mechanical neck pain” OR “non-specific neck pain” OR “mechanical cervical pain” OR “non-specific cervical pain” OR “trigger points in neck region.” We searched the databases from inception until October 2023. Table S2 summarizes details of the literature search.

### 2.2 Inclusion criteria

The population, intervention, comparison, and outcome (PICO) setting of the current meta-analysis was as follows: P: participants with NSNP defined by neck pain without structural or neurological factors [5] and attributed to postural and mechanical origins [13], I: MET defined as a form of osteopathic treatment in which the patient's muscles are actively used on request, from a precisely controlled position, in a specific direction, and against a distinctly executed physician counterforce [7], C: controls in whom MET was not used, and O: changes in pain and disability.

We included RCTs that (a) investigated pain or disability before and after MET, (b) enrolled adults diagnosed with NSNP based on the area of pain or tenderness detected on palpation of the cervical region, and (c) had at least one control group that used treatments other than MET.

### 2.3 Exclusion criteria

Exclusion criteria were as follows: (a) non-RCTs, (b) studies that included patients with cervical radiculopathy/myelopathy/spine fractures, neck surgery, or any systemic disorders that cause neck pain and dysfunction, (c) studies that did not describe pain intensity assessment, and (d) studies that enrolled participants that overlapped with a previously published trial.

### 2.4 Primary outcome measurement

The primary outcome was changes in pain intensity pre- and post-intervention. We confirmed the reliability of scales used in each trial. Modarresi et al. [14] reported that the reliability of the numeric rating scale (NRS) and visual analog scale (VAS) for evaluation of neck pain was 0.58 and 0.93, respectively (based on the intraclass correlation coefficient [ICC]). The McGill Pain Questionnaire (MPQ) is a common tool used to measure pain intensity. Costa et al. [15] observed that the MPQ showed good reliability for evaluation of musculoskeletal pain (ICCs 0.69–0.85). A moderate correlation was observed between the MPQ and NRS scores (ICCs 0.44–0.62).

### 2.5 Secondary outcome measurements

Secondary outcomes were changes in disability measured using the neck disability index (NDI), neck pain, and disability scale (NPAD), or the Northwick Park neck pain questionnaire (NPQ). The ICC of the NDI was 0.64 [16], which indicates moderate reliability. In contrast, ICCs of the NPAD and NPQ were 0.93 [17] and 0.94 [18], respectively, which indicate high reliability. With regard to validity, we observed significant correlations between the NDI and NPAD (r = 0.77) [19] and between the NDI and NPQ (r = 0.89) [20].

### 2.6 Data extraction

Two independent reviewers (L.-H.L. and T.-Y.L.) extracted the following data: demographics, study design, intervention details, outcome values, and measurement timings. The rationale for exclusion of papers was documented. Disagreements between reviewers were resolved through consensus-based discussion or through the intervention of the corresponding author. In cases where data was unavailable or lacked clarity within the published articles, we reached out to the corresponding authors to request the original data or seek additional clarification. With regard to post-treatment data available across multiple time points, the outcome reported at the end of the intervention was included in the analysis. Similar eligible groups were combined for pairwise comparisons in multiple-arm studies [21]. However, in certain three-arm studies in which MET was used as monotherapy, as well as a component of combination therapy, the latter group was excluded from the analysis because the clinical significance of the effects of MET monotherapy was deemed greater than that of its adjuvant effect. Medians and interquartile ranges were converted into means and standard deviations for data pooling based on the Cochrane Handbook for Systematic Reviews of Interventions guidelines [22]. Equations were employed to amalgamate or covert data and the necessary calculations were detailed within Table S3 based on Cochrane handbook for systematic reviews and Tagliaferri et al.’s study [[21], [22], [23]]. To enhance data transparency, we organized all numerical data into an Excel file, included as Supplementary Data 1 for pain and Supplemental Data 2 for disability.

### 2.7 Assessment and quality classification

The Cochrane Risk of Bias tool (RoB 2) was used to assess the quality of the included studies. The RoB 2 consists of the following domains associated with study quality: randomization procedures, adherence to intervention, missing data on outcomes, measurement of outcomes, selective reporting, and overall risk of bias [24]. Intention-to-treat and per-protocol analyses are used for evaluation of intervention adherence. We preferred the per-protocol method because it aligned with the design of most included studies [24].

### 2.8 Statistical analysis

Owing to heterogeneity of the treatment protocols used across the enrolled studies, we used a random effects model to pool effect sizes. The Comprehensive Meta-Analysis software, version 3 (Biostat, Englewood, NJ, US) was used in this study. A two-tailed p value < 0.05 was considered statistically significant. In consideration of the diverse measurement tools employed across the studies included in this meta-analysis, the utilization of the mean difference as a measure was determined to be inappropriate. As a result, we chose to utilize Hedges' g as our preferred standardized effect size measure. This decision was made to ensure the comparability of study outcomes, given the variation in measurement scales used across the included studies. We presented our findings using Hedges' g, accompanied by corresponding 95 % confidence intervals (CIs), in order to quantify and convey the results of the study. Hedges' *g* values of 0.2, 0.5, and 0.8 represented small, moderate, and large effect sizes, respectively [25]. We computed the change scores from pre/post scores. In our study, in the absence of the precise distribution for pre-post change, we would adopt a common approach, assuming a pre-post correlation of 0.5 to address this situation, such as in the article written by Cheung [26]. According to the Cochrane Handbook, the I^2^and Cochran's Q statistic was used to evaluate the degree of heterogeneity across studies. $I^{2}$ values of 25.0 %, 50.0 %, and 75.0 % represented low, moderate, and high heterogeneity, respectively [27].

Sensitivity analyses were performed using the one-study removal method to determine whether removal of a particular trial resulted in a significant change in the summary effect size. Subgroup analyses were performed based on MET regimens and symptom stage (non-chronic ≤3 months; chronic stage >3 months; patients mixed with chronic and non-chronic stages and not-mentioned) administered. Meta-regression was performed to determine whether the pain-alleviating effects of MET correlated with treatment duration. Potential publication bias was evaluated in accordance with the guidelines established by the Cochrane Handbook for Systematic Reviews of Interventions using visual inspection of the funnel plots and hypothesis testing using the Egger's regression test [28].

### 2.9 Assessment of certainty of evidence

The certainty of the evidence was evaluated utilizing the Grading of Recommendations Assessment, Development, and Evaluation (GRADE) tool [29]. The assessment involved categorizing evidence outcomes into four levels: 'high,' 'moderate,' 'low,' or 'very low.' This categorization was based on an analysis of several factors, including the potential for bias, inconsistencies, indirect references, imprecision, and the presence of publication bias. The evaluation process was carried out independently by two reviewers, specifically, L.-H.L. and T.-Y.L. In instances where differences in their assessments arose, efforts were made to facilitate discussions or reach a consensus with the corresponding author.

## 3 Results

### 3.1 Study identification and selection

**Fig. 1:**
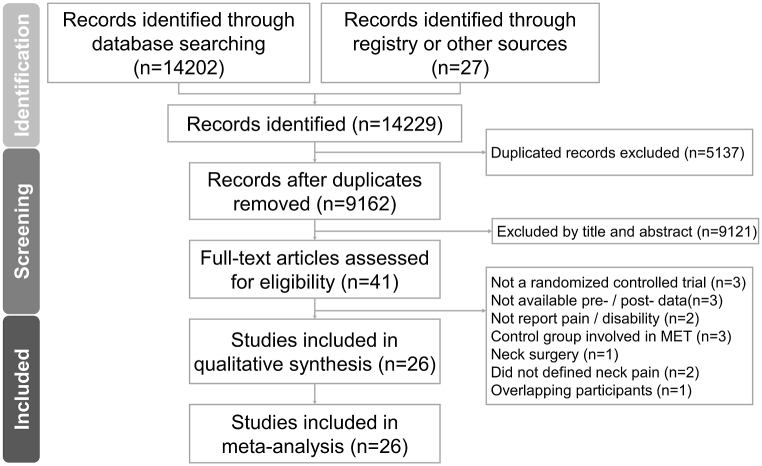
The flow diagram describing the screening and review process.

Fig. 1 shows the PRISMA flowchart for the literature search. Among 1213 non-duplicated citations identified in the literature, 41 articles were further analyzed to confirm their eligibility. We excluded 15 articles after reading the full text; three were non-RCTs, pre- and post-intervention pain/disability assessments were unavailable in three, pain intensity and disability were not reported in two, control groups that did not use MET were unavailable in three, patients without NSNP were recruited in two, patients who underwent modified radical neck dissection were included in one, and participant overlap with another publication included in our meta-analysis was observed in one article. Table 3 summarizes the exclusion criteria.

Finally, 26 (12 three-arm and 14 two-arm) RCTs (1170 participants, mean age 21–49 years, intervention duration 1 day to 10 weeks) were included. Among the three-arm RCTs, one compared MET, low-level laser, and conventional treatment [30], two compared MET, ischemic compression technique, and conventional interventions [31,32], one compared MET plus shock wave and shock wave monotherapy [33], one compared MET, massage plus stretching exercise, and no intervention [34], two compared MET, myofascial release, and routine physical therapy [35,36], one compared MET, ischemic compression and strain counter-strain therapy [37], one compared MET, proprioceptive neuromuscular facilitation, and supervised home exercises [38], one compared MET, MET with dry needling, and dry needling alone [39], one compared MET associated with PIR, MET associated with RI, and conventional interventions [40], one compared MET, deep neck flexor exercise, and conventional interventions [41] and one compared MET, neck stabilization exercise, and neck care education [42].

Among the two-arm RCTs, one compared MET with neck muscle stretching exercise only [43], one with the active release technique [44], one with ischemic compression plus therapeutic ultrasound [45], one with myofascial release combined with cryotherapy and conventional exercise [46], one with myofascial release [47], one with mobilization movement [48], one with ultrasound combined with neck stabilization exercise and proprioceptive neuromuscular facilitation [49], one with ischemic compression plus neck strengthening and stretching exercise [50], one with first rib mobilization plus proprioceptive training (for example, head relocation practice and gaze stability) [51], one with mobilization movement plus conventional therapy (for example, moist heat pack application and neck strengthening) [52], one with neck/scapular endurance and resistance training [53], one with stretching exercise plus infrared therapy [54], and two with physical therapy (for example, neck strengthening and stretching exercise) [55,56].

With regard to pain intensity measurement, three studies used the NRS, 15 used the VAS, and one used the MPQ. With regard to disability estimation, 18 studies used the NDI, one used the NPAD, and one used the NPQ. Table 1, Table 2 summarize the characteristics of the included studies.

**Table 1:** Characteristics of the included studies.

First author, year	Country	Participants (F/M)	Age	Duration of symptom	Population
Ahmed, 2020	KSA	45 (0/45)	MET: 39.4 ± 11.6 Control: 38.55 ± 13.07	NA	Mechanical neck pain with MTrPs
Alghadir, 2020	Arabia	60 (0/60)	MET: 32.30 Control: 32.33	NA	Nonspecific neck pain with MTrPs
Buttagat, 2021	Thailand	45 (30/15)	MET: 21.3 ± 1.29 Control; 21.15 ± 1.67	>3 months	Chronic neck pain associated with MTrPs
El Laithy, 2018	Egypt	30 (18/12)	MET: 32.46 ± 6.54 Control: 34.86 ± 8.39	NA	Mechanical neck pain
Joshi, 2022	India	48	MET: 43.27 ± 0.68 Control: 43.5 ± 1	MET: 11.61 ± 7.47 months Control: 10.55 ± 6.31 months	Chronic non-specific neck pain
Junaid, 2020	Pakistan	60 (32/28)^a^	MET: 32.25 ± 9.56 Control: 32.55 ± 8.35	NA	Acute mechanical neck pain
Kashyap, 2018	India	51 (51/0)^a^	MET: 22.07 ± 4.11 Control: 21.2 ± 3.40	NA	Mechanical neck pain with MTrPs
Khan, 2022	Pakistan	60 (40/20)	MET: 32.4 ± 4.7 Control: 32.4 ± 5.3	2 weeks–6 weeks,	Non-specific neck pain
Kumar, 2015	India	45	NA	NA	MTrPs in upper trapezius
Kumari, 2016	India	45	MET: 31.53 ± 10.06 Control: 34.77 ± 8.47	MET: 4.33 ± 2.46 Control: 4.13 ± 1.1	Chronic mechanical neck pain with MTrPs
Lytras, 2020	Greece	40 (30/10)	MET: 46.8 ± 8.85 Control: 45.8 ± 7.73	>3 months	Chronic mechanical neck pain with MTrP
Manzoor, 2021	Pakistan	56	Total: 36.89 ± 9.28	NA	Non-specific neck pain
Nugraha, 2020	Indonesia	24 (20/4)	MET: 35.42 ± 2.47 Control: 34.75 ± 4.54	NA	Mechanical neck pain
Osama, 2021	Pakistan	78 (52/26)^a^	MET: 40.86 ± 13.14 Control: 43.09 ± 8.55	At least 4–12 weeks	Neck pain
Phadke, 2016	India	56 (33/23)	MET: 31.78 ± 1.76 Control: 33.22 ± 1.71	4–12 weeks	Mechanical neck pain
Revathy, 2016	India	30	NA	NA	MTrPs in upper trapezius
Sachdeva, 2019	India	40	MET: 37.50 ± 8.88 Control: 38.10 ± 9.06	3 weeks to 6 months	Mechanical neck pain
Sadria, 2017	Iran	64 (32/32)	MET: 28.19 ± 9.77 Control: 27.06 ± 8.54	Minimum of 4 months	MTrPs in upper trapezius
Sata, 2012	India	52	MET: 29.44 ± 5.38 Control: 27.06 ± 8.54	3 weeks to 3 months	MTrPs in upper trapezius
Shadmehr, 2022	Iran	54 (33/21)^a^	MET: 27.60 ± 6.85 Control: 31.10 ± 6.65	NA	MTrPs in upper trapezius
Shady, 2021	Egypt	40 (27/13)	MET: 26.40 ± 3.25 Control: 26.40 ± 3.62	MET: 5.45 ± 2.60 months Control: 4.95 ± 2.09 months	Chronic mechanical neck pain
Shah, 2015	India	30 (19/11)	MET: 33.2 ± 3.61 Control: 35.66 ± 5.32	<3month	Non-specific neck pain with MTrP
Tank, 2018	India	40	NA	<3month	Mechanical neck pain
Yadav, 2015	India	33 (15/18)	MET: 32.18 ± 5.25 Control: 33.09 ± 4.57	Minimum duration of 6 weeks	Mechanical neck pain
Yeganeh Lari, 2016	Iran	60 (60/0)	MET: 24.78 ± 0.72 (SE) Control: 24.60 ± 0.93 (SE)	NA	MTrPs in upper trapezius
Zibiri, 2019	Nigeria	35 (23/12)	MET: 49.50 ± 17.50 Control: 45.64 ± 13.29	>3 month	Chronic mechanical neck pain

KSA, Kingdom of Saudi Arabia; MET, muscle energy technique; NA, not available; MTrPs, myofascial trigger points; SE, standard error. Age and pain duration are presented as mean ± standard deviation or range.

a Allocated participants.

**Table 2:** Summary of the intervention details of the included trials.

First author, year	MET group (per-protocol N)	Control group (per-protocol N)	MET treatment protocol	Outcome measurement
Ahmed, 2020	MET + conventional treatment (15)	Laser + conventional treatment (30)	Total intervention period: 5 days MET: 5-s hold, 3-s relaxation by exhalation while reaching the new barrier (upper trapezius) (moderate isometric contraction approximately 75 % of MVC), 3 times/session, 5 sessions/week	Pain: VAS Disability: NDI
Alghadir, 2020	MET + ischemic compression + conventional intervention (40)	Conventional interventions (20)	Total intervention period: 1 day MET: 5-s hold, 3-s relaxation by exhalation while reaching the new barrier (upper trapezius) (moderate isometric contraction approximately 75 % of MVC), 4 times	Pain: VAS
Buttagat, 2021	MET only (15)	Thai massage + stretch exercise + No intention (30)	Total intervention period: 2 weeks MET: 7-s hold + 30-s passive stretch while reaching the new barrier (neck extensor) (mild isometric contraction approximately 20 % of MVC), 3 times/session, 4 sessions/week,	Pain: VAS Disability: NDI
El Laithy, 2018	MET + traditional physical therapy program (15)	Traditional physical therapy program (15)	Total intervention period: 4 weeks MET: 7-s hold, 3-s relaxation by exhalation while reaching the new barrier + 30-s stretch while reaching the new barrier (upper trapezius, levator scapulae, sternocleidomastoid, sternocleidomastoid and suboccipital muscles) (NA isometric contraction approximately NA % of MVC), 3 times/sessions, 3 sessions/week	Disability: NPAD
Joshi, 2022	MET + posture correction exercise (23)	Conventional exercise program (25)	Total intervention period: 3 weeks MET: 7-s hold + 10-s passive stretch while reaching the new barrier (pectoral minor) (mild isometric contraction approximately 20 % of MVC), 3 times/session, NA sessions/week	Pain: NPRS Disability: NDI
Junaid, 2020	MET + routine physical therapy (20)	Myofascial release + routine physical therapy (40)	Total intervention period: 2 weeks MET: (NA isometric contraction approximately NA of MVC), 3 session/week, NA times/session	Pain: NPRS Disability: NDI
Kashyap, 2018	MET + conventional interventions (15)	Manual pressure release + conventional interventions (30)	Total intervention period: 5 days MET: 5-s hold, 3-s stretch while reaching the new barrier (upper trapezius) (moderate isometric contraction approximately 75 % of MVC), 4 times/sessions, NA total session	Pain: VAS Disability: NDI
Khan, 2022	MET + cryotherapy + neck isometric strengthening exercises (30)	Myofascial release + cryotherapy + neck isometric strengthening exercises (30)	Total intervention period: 2 weeks MET: 10-s hold, 10-s stretch while reaching the new barrier (upper trapezius and levator scapulae) (NA isometric contraction approximately NA of MVC), 5 times/sessions, 3 sessions/week	Pain: VAS Disability: NDI
Kumar, 2015	MET + TENS (15)	Ischemic compression + TENS + Strain counter-strain (30)	Total intervention period: 4 weeks MET: 7-10-s hold + 30-s passive stretch while reaching the new barrier (upper trapezius) (mild isometric contraction approximately 20 % of MVC), 5 times/sessions, 3 sessions/week	Pain: VAS Disability: NDI
Kumari, 2016	MET + supervised & home exercise program (15)	PNF + supervised and home exercise program (30)	Total intervention period: 4 weeks MET: 7-10-s hold + 30-s stretch while reaching the new barrier (upper trapezius and levator scapulae) (mild isometric contraction approximately 20 % of MVC), 3–5 times/session, 3 sessions/week	Pain: VAS Disability: NDI
Lytras, 2020	MET + neck and scapula muscles exercise + IC + strain-counterstrain (20)	Neck and scapula muscles exercise (20)	Total intervention period: 10 weeks MET: 7-10-s hold + 30-s passive stretch while reaching the new barrier (levator scapulae, upper trapezius, splenius capitis, and sternocleidomastoid muscles) (mild isometric contraction approximately 20 % of MVC), 3 times/session, 4 sessions/week	Pain: VAS Disability: NDI
Manzoor, 2021	MET only (28)	Mobilization with movement (28)	Total intervention period: 3 weeks MET: 5-10-s hold, 30-s stretch while reaching the new barrier (sternocleidomastoid and upper trapezius muscles) (NA isometric contraction approximately NA of MVC), 5 times/session, 2 sessions/week	Pain: VAS Disability: NDI
Nugraha, 2020	MET + ultrasound therapy + PNF (12)	Ultrasound therapy, neck stabilization exercise, and PNF (12)	Total intervention period: NA MET: NA -s hold, 20 -s stretch while reaching the new barrier (upper trapezius and levator scapulae) (mild isometric contraction approximately 20 % of MVC), 5 sessions/weeks	Pain: VAS Disability: NPNPQ
Osama, 2021	MET + TENS + hot pack + mobilization (49)	TENS + hot pack + mobilization + stretch (22)	Total intervention period: 5 sessions (days) MET: 7-10-s hold, followed by rest period of 5-s10-60-s stretch while reaching the new barrier (anterior, middle, and posterior scalenius, sternocleidomastoid, levator scapulae and upper fibers of the trapezius muscle) (mild isometric contraction approximately 30–50 % of MVC), 3–5 times/session	Pain: NPRS
Phadke, 2016	MET only (28)	Stretch (28)	Total intervention period: 6 sessions (days) MET: NA-s hold, 20-s stretch while reaching the new barrier (levator scapulae and upper trapezius muscle) (mild isometric contraction approximately 20 % of MVC), 3–5 times/session	Pain: VAS Disability: NDI
Revathy, 2016	MET + Ultrasound (15)	Ischemic compression + Ultrasound (15)	Total intervention period: 4 weeks MET: 7-10-s hold + NA-s stretch while reaching the new barrier (upper trapezius) (NA isometric contraction approximately NA of MVC), NA times/session, NA sessions/week	Pain: VAS
Sachdeva, 2019	MET + proprioceptive training (20)	First rib mobilization + proprioceptive training (20)	Total intervention period: 4 weeks MET: 5-7-s hold, NA-s stretch while reaching the new barrier (scalene) (mild isometric contraction approximately NA of MVC), three sessions per week for four weeks	Pain: MPQ Disability: NDI
Sadria, 2017	MET only (32)	ART (32)	Total intervention period: 1 week MET: 7-10-s hold, 30 -s stretch while reaching the new barrier (upper trapezius muscle) (mild isometric contraction approximately 20 % of MVC), 2–3 times/session	Pain: VAS
Sata, 2012	MET only (25)	Myofascia release (27)	Total intervention period: 6 days MET: 10 -s hold, 10 -s stretch while reaching the new barrier (levator scapulae and upper trapezius muscle) (mild isometric contraction approximately 30 % of MVC), 3–4 times/session, 1 session/day	Pain: VAS Disability: NDI
Shadmehr, 2022	MET only (18)	Shock wave (18)	Total intervention period: 1 week MET: 7-10-s hold + 30-s passive stretch while reaching the new barrier (upper trapezius) (mild isometric contraction approximately 20–50 % of MVC), 5 times/session, 3 sessions/week	Pain: VAS Disability: NDI
Shady, 2021	MET + IR (20)	Stretch exercise + IR (20)	Total intervention period: 4 weeks MET: 7-s hold, 7-s relaxation by exhalation while reaching the new barrier, 30-s stretch (upper trapezius, levator scapulae and sternocleidomastoid muscles) (mild isometric contraction approximately 20 % of MVC), 3 times/session, 3 sessions/week	Pain: VAS Disability: NDI
Shah, 2015	MET + neck isometric exercise + stretch (15)	Ischemic compression + isometric neck exercise + stretch (15)	Total intervention period: 1 week MET: 7-10-s hold, NA-s stretch while reaching the new barrier (upper trapezius muscle) (mild isometric contraction approximately NA of MVC), 2–3 times/session	Pain: VAS
Tank, 2018	MET + conventional therapy (20)	Mobilization with movement + conventional therapy (20)	Total intervention period: 2 weeks MET: 5-7-s hold, NA-s stretch while reaching the new barrier (NA) (mild isometric contraction approximately NA of MVC), 2–3 times/session, 6 sessions/week	Pain: VAS Disability: NDI
Yadav, 2015	MET + conventional therapy (11)	Conventional treatment + deep neck flexors training (22)	Total intervention period: 2 weeks MET: 7-10-s hold, NA-s stretch while reaching the new barrier (upper trapezius, levator scapulae and scalene) (mild isometric contraction approximately 20 % of MVC), 3–5 times/session, 5 sessions/week	Pain: VAS Disability: NDI
Yeganeh Lari, 2016	MET only (20)	Dry needle (20)	Total intervention period: 1 week MET: 7–10s hold + 30-s passive stretch while reaching the new barrier (upper trapezius) (mild isometric contraction approximately 20 % of MVC), 3–5 times/session, 3 sessions/week	Pain: VAS
Zibiri, 2019	MET + neck care education + IR (12)	Neck stabilization exercise + neck care education + IR (23)	Total intervention period: 8 weeks MET: 5-s hold, 10-s stretch while reaching the new barrier (NA isometric contraction approximately NA % of MVC), 3 times/session, 2 sessions/week	Pain: VAS Disability: NDI

ART, active release technique; IR, infrared radiation; MET, muscle energy technique; MPQ, Mcgill Pain Questionnaire; N, number; NA, non-assessment; NDI, neck disability; NPRS, Numeric Pain Rating Scale; NPAD, Neck Pain and Disability Scale; NPNPQ, Northwick Park Neck Pain Questionnaire; TENS, transcutaneous electrical nerve stimulator; PNF, proprioceptive neuromuscular facilitation; VAS, Visual Analogue Scale.

**Table 3:** Detailed quality assessment of included studies using Cochrane risk of bias 2 tool.

First Author	Year	Randomization process	Intervention adherence	Missing outcome data	Outcome measurement	Selective reporting	Overall RoB
Ahmed	2020	L	L	L	L	L	L
Alghadir	2020	L	L	L	L	L	L
Buttagat	2021	S^1^	L	L	L	L	S
EI Laithy	2018	L	L	L	L	L	L
Joshi	2022	H^2,3^	L	L	L	L	H
Junaid	2020	L	L	S^4^	L	L	S
Kashyap	2018	L	L	S^5^	L	L	S
Khan	2022	L	L	L	L	L	L
Kumar	2015	H^3,6^	L	H^7^	L	L	H
Kumari	2016	S^3^	L	L	L	L	S
Lytras	2020	L	L	L	L	L	L
Manzoor	2021	S^6^	L	L	L	L	S
Nugraha	2020	L	L	H^7^	L	L	H
Osama	2021	L	L	S^8^	L	L	S
Phadke	2016	L	L	S^9^	L	L	S
Revathy	2016	H^3,6^	L	H^7^	L	L	H
Sachdeva	2019	L	L	L	L	L	L
Sadria	2017	L	L	L	L	L	L
Sata	2012	S^6^	L	L	L	L	S
Shadmehr	2022	L	L	L	L	L	L
Shady	2021	L	L	H^7^	L	L	H
Shah	2015	S^6^	L	L	L	L	S
Tank	2018	S^6^	L	H^7^	L	L	H
Yadav	2015	L	L	H^7^	L	L	H
Yeganeh Lari	2016	S^3^	L	H^7^	L	L	H
Zibiri	2019	L	L	S^5^	L	L	S

^1^There was a significant difference in the age between groups.

^2^There was a significant difference between experimental and control groups in pain intensity at baseline.

^3^There was no reported proper allocation concealment.

^4^Twenty-seven subjects discontinued intervention and missed assessment.

^5^Six subjects discontinued intervention and missed assessment.

^6^There was no data on the demographic or baseline of participants.

^7^There was no information about the extent of missing outcome data.

^8^Seven subjects discontinued intervention and missed assessment.

^9^Four subjects discontinued intervention and missed assessment.

### 3.2 Methodological quality of the included studies

With regard to methodological quality of the included studies, 30.8 % of the studies had a low risk of bias, 38.4 % (n = 10) had some risk of bias owing to missing outcomes and 30.8 % had a high risk of bias (n = 8) (Fig. S1). The high risk of bias in most RCTs was associated with missing outcome data, followed by bias associated with the randomization process. Table 3 summarizes details of the risk of bias assessment.

### 3.3 Assessment of evidence certainty

We further assessed the meta-results of GRADE certainty evidence. The certainty of evidence for over all pain intensity and disability was considered “low”. The certainty of evidence for subgroup analysis pain intensity and disability was considered “moderate to low”. Detailed results can be found in Table S4."

### 3.4 Date handling

Overall, data extraction methods were reported in 26 trials as summarized in the following manner. Among these trials, 24 trials extracted the mean and standard deviation data directly from tables. In one trial, the median and interquartile range were extracted from a table and subsequently converted using formulas. In one trial, the standard error of the mean was initially obtained from a table and then converted to standard deviation for pooled analysis. Additionally, ten trials involved two comparator arms or two MET arms, and their data were pooled for the respective analyses. For more detailed information regarding the specific data handling procedures, please refer to Table S5.

### 3.5 Effectiveness of the muscle energy technique in pain reduction

**Fig. 2:**
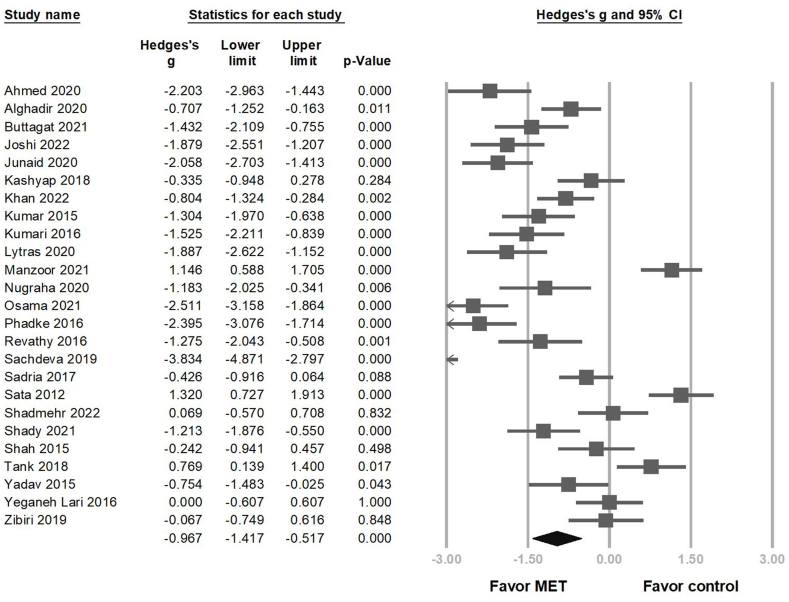
Forest plot of the overall effects of muscle energy technique (MET) on pain reduction in patients with non-specific neck pain.

**Fig. 3:**
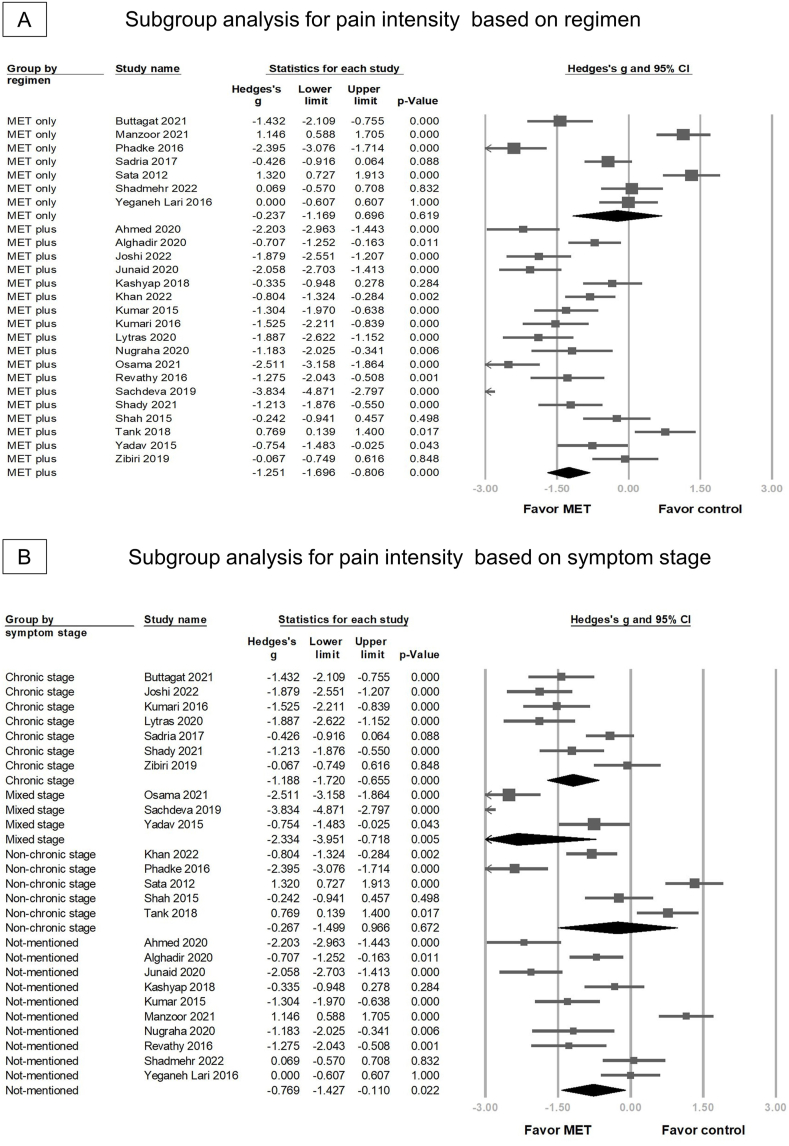
The forest plot of subgroup analysis for pain intensity (A) based on regimen of muscle energy technique (MET) and (B) symptom stage of non-specific neck pain (NSNP).

Overall, 25 RCTs reported significant reduction in pain intensity in the MET group (Hedges' *g* = −0.967 95 % CI = −1.417 to −0.517, *p* < 0.001, $I^{2}$ = 91.588 %) (Fig. 2). Sensitivity analysis using the one-study removal method showed a consistently significant effect of MET on pain reduction (Fig. S2). Subgroup analysis was performed based on intervention regimens and symptom stage. We observed significant pain reduction in the group that used MET combined with other treatments (for example, neck/scapular strengthening exercises, or myofascial release) (Hedges' *g* = −1.251, 95 % CI = −1.696 to −0.806, *p* < 0.001) (Fig. 3A). However, MET monotherapy did not provide significant pain relief (Hedges' *g* = −0.237, 95 % CI = −1.169 to 0.696, *p* = 0.619). In the symptom stage group, the substantial overlap of the 95 % confidence intervals for the effect sizes in all four groups indicates that there were no significant differences in the effect sizes among these groups (chronic stage: Hedges' g = −1.188, 95 % CI = −1.721 to −0.655; non-chronic stage: Hedges' g = −0.627, 95 % CI = −1.499 to −0.966; patients mixed with chronic and non-chronic stages: Hedges' g = −2.334, 95 % CI = −3.951 to −0.718; not-mentioned: Hedges’ g = −0.769, 95 % CI = −1.427 to −0.110) (Fig. 3B).

Meta-regression analysis was performed to investigate whether treatment duration (24 RCTs) and frequency of MET sessions per week (16 RCTs) modified the effects on pain reduction. The regression coefficient for treatment duration (days) was −0.035 (95 % CI −0.055 to −0.015, *p* < 0.001) and for the session frequency per week was −0.224 (95 % CI −0.377 to −0.071, *p* = 0.004), which indicates that increased treatment duration/frequency contributed to greater pain reduction (Figs. S3 and S4).

### 3.6 Effectiveness of muscle energy technique in reduction of disability

**Fig. 4:**
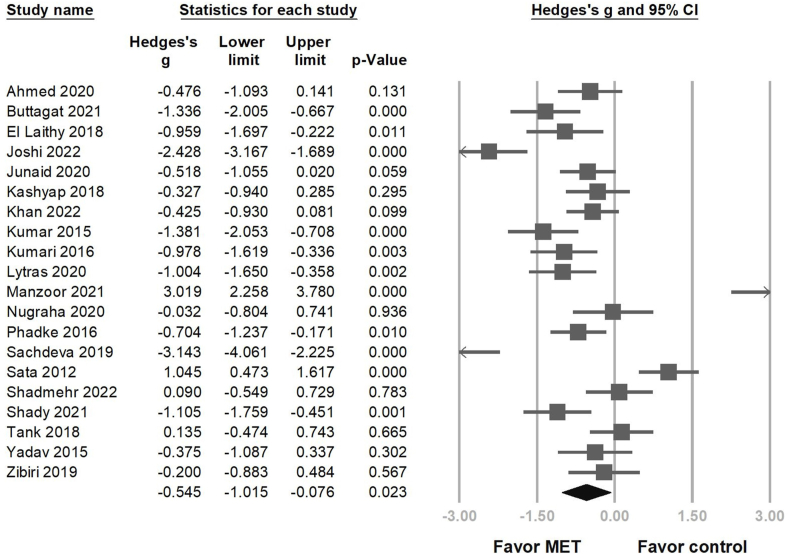
Forest plot of the overall effects of muscle energy technique (MET) on disability in patients with non-specific neck pain.

**Fig. 5:**
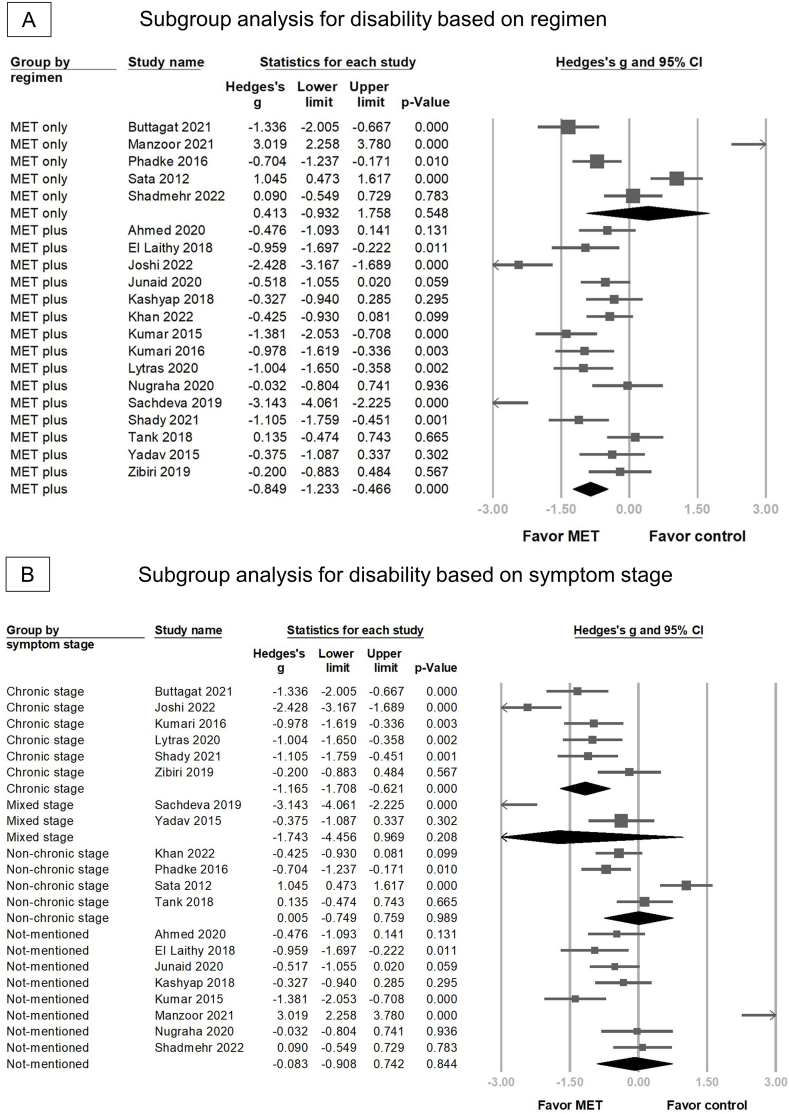
The forest plot of subgroup analysis for disability (A) based on regimen of muscle energy technique (MET) and (B) symptom stage of non-specific neck pain (NSNP).

Compared with the control group, the MET group showed significantly reduced disability post intervention in 20 RCTs (Hedges' *g* = −0.545, 95 % CI = −1.015 to − 0.076, *p* = 0.023, $I^{2}$ = 91.025) (Fig. 4). Similarly, sensitivity analysis using the one-study removal method showed a significant consistent effect of MET on disability (Fig. S5). MET combined with other treatments significantly reduced disability (Hedges' *g* = −0.849, 95 % CI = −1.233 to −0.466, *p* < 0.001) (Fig. 5A); however, this benefit was not significant in the MET monotherapy group (Hedges' *g* = 0.413, 95 % CI = −0.932 to 1.758, *p* = 0.548). In the group characterized by symptom stage, there is substantial overlap in the 95 % CIs for the effect sizes in all four subgroups. This suggests that there were no statistically significant differences in the effect sizes among these groups. Specifically, for the chronic stage, the Hedges’ *g* was −1.165 (95 % CI = −1.708 to −0.621); for the non-chronic stage, it was 0.005 (95 % CI = −0.749 to 0.759); for patients mixed with chronic and non-chronic stages, it was −1.743 (95 % CI = −4.456 to 0.969); and for the stage not-mentioned, it was −0.083 (95 % CI = −0.908 to 0.742) (Fig. 5B).

Meta-regression analysis showed a regression coefficient of −0.022 (95 % CI, −0.040 to −0.005, *p* = 0.013) for treatment duration and −0.170 (95 % CI, −0.335 to −0.005, *p* = 0.043) for session frequency, which indicates that increased treatment duration/frequency contributed to greater disability improvement (Figs. S6 and S7).

### 3.7 Publication bias

The funnel plot for pain intensity revealed asymmetry in the distribution of effect sizes (Fig. S8), with a *p* value of 0.007 using the Egger's regression test. Nevertheless, the effect size distribution for disability (Fig. S9) showed no asymmetry, with a *p* value of 0.381 using the Egger's regression test.

## 4 Discussion

To our knowledge, this is the first meta-analysis of RCTs that investigated the effectiveness of MET in reducing pain and disability in patients with NSNP. We observed that MET resulted in significant reduction in pain and disability; however, the benefits were more pronounced with MET used in conjunction with other treatments in contrast to MET monotherapy.

Our meta-analysis showed that pain relief and reduction in disability were significantly better in the MET than in the control group. However, these effects cannot be solely attributed to muscle relaxation, and several other pathways, such as stimulation of the pain-inhibiting cascade, likely contribute to its effectiveness. For example, MET involves isometric contraction of agonist muscles, which inhibits the Golgi tendon reflex and causes consequent reflex relaxation of the antagonist muscles. This process also activates mechanoreceptors on joints and muscles and results in excitation of the sympathetic system via somatic afferents and activation of the periaqueductal gray matter, which regulates descending pain modulation [57]. Additionally, rhythmic muscle contraction during MET affects the rate of lymphatic and blood flow, which alters the interstitial pressure and improves transcapillary blood flow, which can desensitize peripheral nociceptive chemical mediators such as cytokines [58].

Our results highlight that MET used in combination with other treatments provided better pain and disability relief than that associated with MET monotherapy. This finding may be attributable to the following factors: (A) Most trials that used MET monotherapy included patients diagnosed with MTrP in the neck region, and the control groups in these studies received treatments such as dry needling, shock wave, myofascial release, active release technique, and massage, which confirms the therapeutic effectiveness of MET in MTrP. (B) Treatment duration/sessions may have contributed to the differences in outcomes observed between groups that received MET as combination therapy vs. MET monotherapy. Treatment duration was likely longer and treatment sessions were more frequent than those used in the latter group (Table 2). Meta-regression analysis showed that longer and more frequent sessions were associated with greater pain relief and reduced disability. (C) Several factors, such as poor posture and muscle control affect NSNP. The upper crossed syndrome characterized by muscular imbalances in the neck and shoulder girdle is a common postural abnormality associated with NSNP. This syndrome can lead to tightness in the upper trapezius, pectoralis, and levator scapulae muscles and weakness in the rhomboids, serratus anterior, middle and lower trapezius, and deep neck flexors [59]. Although MET monotherapy can improve muscle extensibility, it cannot satisfactorily address muscle weakness associated with NSNP, including lower trapezius weakness. Strengthening the lower trapezius improves postural alignment and reduces the intensity of neck pain and neck disability, as described by Park et al. [60]. Therefore, combination therapy comprising MET and other exercise interventions such as lower trapezius strengthening enhances treatment effectiveness.

Among the 26 RCTs included in the study, no study reported adverse events, which is consistent with the findings of previous systematic reviews that have investigated MET [10]. However, some precautions are warranted when performing MET, particularly in the vicinity of fractures and areas of severe osteoporosis/arthritis [8]. Batavia [61] suggested that most concerns regarding MET are either procedural or associated with underlying musculoskeletal pathologies. Procedural concerns are associated with the use of overly aggressive techniques, particularly those performed by inexperienced practitioners. Musculoskeletal concerns including tissue fragility or joint hypermobility are also relevant in patients who receive combination therapy that involves joint manipulation. Therefore, comprehensive evaluation is essential to accurately identify the aforementioned concerns, if any, before performing the MET.

Following are the limitations of this study: (i) We observed significant heterogeneity in the overall effect sizes for pain and disability. To address this issue, we performed subgroup analyses based on the MET regimens and symptom stages to identify potential contributors to heterogeneity. (ii) The follow-up duration in most included studies was short, which precluded assessment of the long-term effects of MET on reduction of pain and disability. Therefore, further studies with longer follow-up are warranted. (iii) Our meta-analysis revealed publication bias in the effect size of pain intensity. An updated meta-analysis should be performed in future to confirm whether the current publication bias may have affected the effects of MET observed in this study. (iv) Prediction intervals in meta-analysis estimate the potential range of effect sizes for studies. They consider uncertainty, heterogeneity, and provide a broader perspective than confidence intervals, which measure precision. Prediction intervals are valuable for planning studies, acknowledging uncertainty, and handling heterogeneity, especially in cases with few studies [62]. They offer a more comprehensive view of potential effect sizes [63]. However, it's important to note that our current meta-analysis software, Comprehensive Meta-Analysis V3, lacks the capability to calculate prediction intervals. To confirm the prediction interval, future meta-analyses with updated software should be conducted.

In conclusion, the evidence was of low certainty in our meta-analysis and demonstrated that MET seemed to be effective when incorporated into a combined treatment approach for NSNP. This combined approach addresses the multifaceted nature of NSNP and might provide better pain relief and disability improvement. Future studies should prioritize conducting higher-quality clinical controlled trials, extending follow-up durations, and presenting prediction intervals.

## Funding

This study was funded by the 10.13039/501100005762National Taiwan University Hospital, Bei-Hu Branch; 10.13039/501100004663Ministry of Science and Technology, Taiwan (MOST 106-2314-B-002-180-MY3 and MOST 109-2314-B-002-114-MY3) and National Science and Technology, Taiwan (NSTC 112-2314-B-002-134).

## Data availability statement

All data generated or analyzed during this study are included in this published article.

## CRediT authorship contribution statement

**Long-Huei Lin:** Writing – original draft, Investigation, Formal analysis, Data curation, Conceptualization. **Ting-Yu Lin:** Writing – original draft, Investigation. **Ke-Vin Chang:** Writing – review & editing, Methodology, Funding acquisition. **Wei-Ting Wu:** Formal analysis. **Levent Özçakar:** Writing – review & editing, Validation, Supervision, Conceptualization.

## Declaration of competing interest

The authors declare the following financial interests/personal relationships which may be considered as potential competing interests:Ke-Vin Chang reports financial support was provided by 10.13039/501100004663Ministry of Science and Technology, Taiwan.
