# Supplementary material for: Muscle energy technique to reduce pain and disability in cases of non-specific neck pain: A systematic review and meta-analysis of randomized controlled trials
Source: Heliyon. 2023 Nov 17;9(11):e22469. doi: 10.1016/j.heliyon.2023.e22469 (PMC10687238; doi:10.1016/j.heliyon.2023.e22469)

**Supplemental Material**

**Table S1.** PRISMA Checklist

| **Section**  **and Topic** | **#** | **Checklist item** | **Location** |
| --- | --- | --- | --- |
| **TITLE** | | |  |
| Title | 1 | Identify the report as a systematic review. | Title |
| **ABSTRACT** | | |  |
| Abstract | 2 | See the PRISMA 2020 for Abstracts checklist. | Abstract |
| **INTRODUCTION** | | |  |
| Rationale | 3 | Describe the rationale for the review in the context of existing knowledge. | Introduction |
| Objectives | 4 | Provide an explicit statement of the objective(s) or question(s) the review addresses. | Introduction |
| **METHODS** | | |  |
| Eligibility criteria | 5 | Specify the inclusion and exclusion criteria for the review and how studies were grouped for the syntheses. | Methods |
| Information sources | 6 | Specify all databases, registers, websites, organisations, reference lists and other sources searched or consulted to identify studies. Specify the date when each source was last searched or consulted. | Methods |
| Search strategy | 7 | Present the full search strategies for all databases, registers and websites, including any filters and limits used. | Methods, Table S2 |
| Selection process | 8 | Specify the methods used to decide whether a study met the inclusion criteria of the review, including how many reviewers screened each record and each report retrieved, whether they worked independently, and if applicable, details of automation tools used in the process. | Methods |
| Data collection process | 9 | Specify the methods used to collect data from reports, including how many reviewers collected data from each report, whether they worked independently, any processes for obtaining or confirming data from study investigators, and if applicable, details of automation tools used in the process. | Methods |
| Data items | 10a | List and define all outcomes for which data were sought. Specify whether all results that were compatible with each outcome domain in each study were sought (e.g., for all measures, time points, analyses), and if not, the methods used to decide which results to collect. | Methods |
|  | 10b | List and define all other variables for which data were sought (e.g., participant and intervention characteristics, funding sources). Describe any assumptions made about any missing or unclear information. | Methods  Table 1 |
| Study risk of bias assessment | 11 | Specify the methods used to assess risk of bias in the included studies, including details of the tool(s) used, how many reviewers assessed each study and whether they worked independently, and if applicable, details of automation tools used in the process. | Methods |
| Effect measures | 12 | Specify for each outcome the effect measure(s) (e.g., risk ratio, mean difference) used in the synthesis or presentation of results. | Methods |
| Synthesis methods | 13a | Describe the processes used to decide which studies were eligible for each synthesis (e.g., tabulating the study intervention characteristics and comparing against the planned groups for each synthesis (item #5)). | Methods, Figure 1,  Table 1, Table S3 |
|  | 13b | Describe any methods required to prepare the data for presentation or synthesis, such as handling of missing summary statistics, or data conversions. | Methods |
|  | 13c | Describe any methods used to tabulate or visually display results of individual studies and syntheses. | Methods |
|  | 13d | Describe any methods used to synthesize results and provide a rationale for the choice(s). If meta-analysis was performed, describe the model(s), method(s) to identify the presence and extent of statistical heterogeneity, and software package(s) used. | Methods |
|  | 13e | Describe any methods used to explore possible causes of heterogeneity among study results (e.g., subgroup analysis, meta-regression). | Methods |
|  | 13f | Describe any sensitivity analyses conducted to assess robustness of the synthesized results. | Methods |
| Reporting bias assessment | 14 | Describe any methods used to assess risk of bias due to missing results in a synthesis (arising from reporting biases). | Methods, Figure S1,  Table 3 |
| Certainty assessment | 15 | Describe any methods used to assess certainty (or confidence) in the body of evidence for an outcome. | Methods |
| **RESULTS** | | |  |
| Study selection | 16a | Describe the results of the search and selection process, from the number of records identified in the search to the number of studies included in the review, ideally using a flow diagram. | Results, Figure 1,  Table S2-S3 |
|  | 16b | Cite studies that might appear to meet the inclusion criteria, but which were excluded, and explain why they were excluded. | Results, Table S3 |
| Study characteristics | 17 | Cite each included study and present its characteristics. | Results, Table 1 |
| Risk of bias | 18 | Present assessments of risk of bias for each included study. | Figure S1, Table 2 |
| Results of individual studies | 19 | For all outcomes, present, for each study: (a) summary statistics for each group (where appropriate) and (b) an effect estimates and its precision (e.g., confidence/credible interval), ideally using structured tables or plots. | Figure 2-5, Figure S2-S9 |
| Results of syntheses | 20a | For each synthesis, briefly summarise the characteristics and risk of bias among contributing studies. | Results, Table 3 |
|  | 20b | Present results of all statistical syntheses conducted. If meta-analysis was done, present for each the summary estimate and its precision (e.g., confidence/credible interval) and measures of statistical heterogeneity. If comparing groups, describe the direction of the effect. | Results, Figure 2-5, Figure S2-S9 |
|  | 20c | Present results of all investigations of possible causes of heterogeneity among study results. | Results, Figure 2-5, Figure S2-S9 |
|  | 20d | Present results of all sensitivity analyses conducted to assess the robustness of the synthesized results. | Results, Figure S2  Figure S5 |
| Reporting biases | 21 | Present assessments of risk of bias due to missing results (arising from reporting biases) for each synthesis assessed. | Figure S1, Table 3 |
| Certainty of evidence | 22 | Present assessments of certainty (or confidence) in the body of evidence for each outcome assessed. | Figure 2-5, Figure S2-S9 |
| **DISCUSSION** | | |  |
| Discussion | 23a | Provide a general interpretation of the results in the context of other evidence. | Discussion |
|  | 23b | Discuss any limitations of the evidence included in the review. | Discussion |
|  | 23c | Discuss any limitations of the review processes used. | Discussion |
|  | 23d | Discuss implications of the results for practice, policy, and future research. | Discussion |
| **OTHER INFORMATION** | | |  |
| Registration and protocol | 24a | Provide registration information for the review, including register name and registration number, or state that the review was not registered. | Methods |
|  | 24b | Indicate where the review protocol can be accessed, or state that a protocol was not prepared. | Methods, Table S2-S3 |
|  | 24c | Describe and explain any amendments to information provided at registration or in the protocol. | Methods, Table S2-S5 |
| Support | 25 | Describe sources of financial or non-financial support for the review, and the role of the funders or sponsors in the review. | Funding |
| Competing interests | 26 | Declare any competing interests of review authors. | Conflicts of interest |
| Availability of data, code and other materials | 27 | Report which of the following are publicly available and where they can be found: template data collection forms; data extracted from included studies; data used for all analyses; analytic code; any other materials used in the review. | Results, Table S2-S5 |

- In the study done by Manzoor et al, we found the data presented on the table did not match their corresponding columns. We contacted the corresponding authors for clarification of the clearly identified typos. However, we did not receive a response yet. Therefore, we used the data corrected by ourself for analysis.

**Table S2.** Keywords and search results in different databases

| **Database** | **Keyword** | **Filter** | **Date** | **Results** |
| --- | --- | --- | --- | --- |
| PubMed | (“muscle energy technique” OR “post-isometric relaxation” OR “reciprocal inhibition”) AND (“mechanical neck pain” OR “non-specific neck pain” OR “mechanical cervical pain” OR “non-specific cervical pain” OR “trigger points in neck region”) | Not used | April 6, 2023 | 2635 |
| Cochrane Library | (“muscle energy technique” OR “post-isometric relaxation” OR “reciprocal inhibition”) AND (“mechanical neck pain” OR “non-specific neck pain” OR “mechanical cervical pain” OR “non-specific cervical pain” OR “trigger points in neck region”) | Title Abstract  Keyword | April 6, 2023 | 3403 |
| Clinical Trials .gov | (“muscle energy technique” OR “post-isometric relaxation” OR “reciprocal inhibition”) AND (“mechanical neck pain” OR “non-specific neck pain” OR “mechanical cervical pain” OR “non-specific cervical pain” OR “trigger points in neck region”) | Condition or disease | April 6, 2023 | 16 |
| PEDro | (“muscle energy technique” OR “post-isometric relaxation” OR “reciprocal inhibition”) AND (“mechanical neck pain” OR “non-specific neck pain” OR “mechanical cervical pain” OR “non-specific cervical pain” OR “trigger points in neck region”) | Condition or disease | April 6, 2023 | 25 |
| Embase | (“muscle energy technique” OR “post-isometric relaxation” OR “reciprocal inhibition”) AND (“mechanical neck pain” OR “non-specific neck pain” OR “mechanical cervical pain” OR “non-specific cervical pain” OR “trigger points in neck region”) | Research articles | October 1, 2023 | 8123 |

**Table S3.** Excluded studies and reasons

| **Citations** | **Reasons** |
| --- | --- |
| Jawade, S., Chitale, N., Jr, & Phansopkar, P. (2023). The Effect of Reciprocal Inhibition Techniques on Pain, Range of Motion, and Functional Activities in Patients With Upper Trapezitis. Cureus, 15(2), e34487. https://doi.org/10.7759/cureus.34487 | Not a randomized controlled trial |
| Nambi, G., Sharma, R., Inbasekaran, D., Vaghesiya, A., & Bhatt, U. (2013). Difference in effect between ischemic compression and muscle energy technique on upper trepezius myofascial trigger points: Comparative study. International Journal of Health & Allied Sciences, 2(1), 17-17. https://doi.org/10.4103/2278-344X.110570 | Not a randomized controlled trial |
| Sakshi, N., Suman, M., & Geetanjali, S. (2014). Effect of muscle energy technique and deep neck flexors exercise on pain, disability and forward head posture in patients with chronic neck pain. National Editorial Advisory Board, 8(4), 43. https://doi.org/10.5958/0973-5674.2014.00009.4 | Not a randomized controlled trial |
| Saadat, Z., Hemmati, L., Pirouzi, S., Ataollahi, M., & Ali-Mohammadi, F. (2018). Effects of Integrated Neuromuscular Inhibition Technique on pain threshold and pain intensity in patients with upper trapezius trigger points. Journal of bodywork and movement therapies, 22(4), 937–940. https://doi.org/10.1016/j.jbmt.2018.01.002 | No available data for pre- and post- intervention pain/disability assessment or change in pain/disability score |
| Gilani, M. H. Z., Obaid, S., & Tariq, M. (2018). Comparison between effectiveness of ischemic compression and muscle energy technique in upper trapezius myofascial trigger points. Isra Medical Journal, 10, 230-234. | No available data for pre- and post- intervention pain/disability assessment or change in pain/disability score |
| Mahajan, R., Kataria, C., & Bansal, K. (2012). Comparative effectiveness of muscle energy technique and static stretching for treatment of subacute mechanical neck pain. International Journal of Health and Rehabilitation Sciences, 1(1), 16-21. https://doi.org/10.5455/ijhrs.00000004 | No available data for pre- and post- intervention pain/disability assessment or change in pain/disability score |
| Hadamus, A., Wojda, A., & Białoszewski, D. (2021). Can the sleep quality of patients with chronic neck pain be improved by muscle energy techniques combined with Swedish massage?. Complementary therapies in clinical practice, 44, 101421. https://doi.org/10.1016/j.ctcp.2021.101421 | Did not report pain intensity and disability |
| Jeong, H. M., Shim, J. H., & Suh, H. R. (2017). The passive stretching, massage, and muscle energy technique effects on range of motion, strength, and pressure pain threshold in musculoskeletal neck pain of young adults. Physical therapy rehabilitation science, 6(4), 196-201. https://doi.org/10.14474/ptrs.2017.6.4.196 | Did not report pain intensity and disability |
| Basak, T., Pal, T. K., Sasi, M. M., & Agarwal, S. (2018). A comparative study on the efficacy of ischaemic compression and dry needling with muscle energy technique in patients with upper trapezius myofascial trigger points. Int. J. Health Sci. Res, 8, 74-81. | Lacking a control group not using muscle energy technique |
| Siddiqui, M., Akhter, S., & Baig, A. A. M. (2022). Effects of autogenic and reciprocal inhibition techniques with conventional therapy in mechanical neck pain - a randomized control trial. BMC musculoskeletal disorders, 23(1), 704. https://doi.org/10.1186/s12891-022-05668-0 | Lacking a control group not using muscle energy technique |
| Nagrale, A. V., Glynn, P., Joshi, A., & Ramteke, G. (2010). The efficacy of an integrated neuromuscular inhibition technique on upper trapezius trigger points in subjects with non-specific neck pain: a randomized controlled trial. The Journal of manual & manipulative therapy, 18(1), 37–43. | Lacking a control group not using muscle energy technique |
| Thomas, A., D'Silva, C., Mohandas, L., Pais, S. M. J., & Samuel, S. R. (2020). Effect of Muscle Energy Techniques V/S Active Range of Motion Exercises on Shoulder Function Post Modified Radical Neck Dissection in patients with Head and Neck Cancer - A Randomized Clinical Trial. Asian Pacific journal of cancer prevention : APJCP, 21(8), 2389–2393. https://doi.org/10.31557/APJCP.2020.21.8.2389 | Modified radical neck dissection as inclusion criteria |
| Gillani, S. N., Ain, Q.-, Rehman, S. U., & Masood, T. (2020). Effects of eccentric muscle energy technique versus static stretching exercises in the management of cervical dysfunction in upper cross syndrome: a randomized control trial. JPMA. The Journal of the Pakistan Medical Association, 70(3), 394–398. https://doi.org/10.5455/JPMA.300417 | Inclusion criteria did not define neck pain specifically |
| Shahzad, H., Fatima, A., Ahmad, A., Khan, ZA., Gilani, SA., (2022). Effectiveness of routine physical therapy with and without muscle energy technique in patients with upper crossed syndrome. Rawal Medical Journal, 47(3), 654-657. | Inclusion criteria did not define neck pain specifically |
| Osama, M., & Shakil Ur Rehman, S. (2020). Effects of static stretching as compared to autogenic inhibition and reciprocal inhibition muscle energy techniques in the management of mechanical neck pain: a randomized controlled trial. JPMA. The Journal of the Pakistan Medical Association, 70(5), 786–790. https://doi.org/10.5455/JPMA.9596 | Participants overlapped with another publication of the author (Osama 2020) |

**Table S3** **Calculations from the Cochrane Handbook used in the review and** **Tagliaferri et al’s study**

| **Used for** | **Calculation** |
| --- | --- |
| Standard Error to Standard Deviation | $SD=SE\times\sqrt{N}$ |
| Pooling Mean | $\frac{N1M1+N2M2}{N1+N2}$ |
| Pooling Standard Deviations | $\frac{\left( N1-1 \right){SD1}^{2}+\left( N2-1 \right){SD2}^{2}+\frac{N1M1\times N2M2}{N1+N2}({M1}^{2}+{M2}^{2}-2M1M2)}{N1+N2-1}$ |
| Median to Mean | $Median \approx Mean$ |
| Interquartile Range to Standard Deviation | IQR/1.35 |

**Table S4. Assessment of evidence quality for each outcome**

| **Quality assessment** | | | | | | | **No of patients** | | **Effect** | | **Quality** | **Importance** |
| --- | --- | --- | --- | --- | --- | --- | --- | --- | --- | --- | --- | --- |
|  |  |  |  |  |  |  |  |  |  |  |  |  |
| **No of studies** | **Design** | **Risk of bias** | **Inconsistency** | **Indirectness** | **Imprecision** | **Other considerations** | **MET**  **Control** | | **Relative**  **(95% CI)** | **Absolute** |  |  |
| **Pain intensity overall** | | | | | | | | | | | | |
| 25 | randomised trials | serious^1^ | very serious^2^ | no serious indirectness | no serious imprecision | none | 533 | 607 | Hedges’ g - 0.967 lower (-1.147 to -0.517 lower) |  | LOW | CRITICAL |
| **Pain intensity regimen subgroup analysis ( MET only)** | | | | | | | | | | | | |
| 7 | randomised trials | serious^1^ | very serious^2^ | no serious indirectness | no serious imprecision | none | 166 | 183 | Hedges’ g - 0.237 lower (-1.169 to 0.696 lower) |  | LOW | CRITICAL |
| **Pain intensity regimen subgroup analysis ( MET plus)** | | | | | | | | | | | | |
| 18 | randomised trials | serious^1^ | very serious^2^ | no serious indirectness | no serious imprecision | none | 367 | 424 | Hedges’ g – 1.251 lower (-1.696 to -0.806 lower) |  | LOW | CRITICAL |
| **Pain intensity symptom stage subgroup analysis (chronic stage)** | | | | | | | | | | | | |
| 7 | randomised trials | serious^1^ | very serious^2^ | no serious indirectness | no serious imprecision | none | 137 | 180 | Hedges’ g – 1.188 lower (-1.720 to -0.655 lower) |  | LOW | CRITICAL |
| **Pain intensity symptom stage subgroup analysis (mix stage)** | | | | | | | | | | | | |
| 3 | randomised trials | serious^1^ | very serious^2^ | no serious indirectness | no serious imprecision | none | 80 | 64 | Hedges’ g – 2.334 lower (-3.951to -0.718 lower) |  | LOW | CRITICAL |
| **Pain intensity symptom stage subgroup analysis (Non-chronic stage)** | | | | | | | | | | | | |
| 5 | randomised trials | serious^1^ | very serious^2^ | no serious indirectness | no serious imprecision | none | 118 | 120 | Hedges’ g – 0.267 lower (-1.499to 0.966 lower) |  | LOW | CRITICAL |
| **Disability over all** | | | | | | | | | | | | |
| 20 | randomised trials | serious^1^ | very serious^2^ | no serious indirectness | no serious imprecision | none | 377 | 498 | Hedges’ g -0.545 lower (-1.015 to -0.076 lower) |  | LOW | IMPORTANT |
| **Disability regimen subgroup analysis ( MET only)** | | | | | | | | | | | | |
| 5 | randomised trials | No serious bias | very serious^2^ | no serious indirectness | no serious imprecision | none | 144 | 131 | Hedges’ g 0.413 lower (-0.932 to 1.758 lower) |  | Moderate | IMPORTANT |
| **Disability regimen subgroup analysis ( MET plus)** | | | | | | | | | | | | |
| 15 | randomised trials | serious^1^ | very serious^2^ | no serious indirectness | no serious imprecision | none | 233 | 367 | Hedges’ g -0.849 lower (-1.233 to -0.466 lower) |  | LOW | IMPORTANT |
| **Disability symptom stage subgroup analysis (chronic stage)** | | | | | | | | | | | | |
| 6 | randomised trials | serious^1^ | very serious^2^ | no serious indirectness | no serious imprecision | none | 117 | 160 | Hedges’ g -1.165  lower (-1.708 to -0.621 lower) |  | LOW | IMPORTANT |
| **Disability symptom stage subgroup analysis (mixed stage)** | | | | | | | | | | | | |
| 2 | randomised trials | serious^1^ | very serious^2^ | no serious indirectness | no serious imprecision | none | 69 | 42 | Hedges’ g -1.743  lower (-4.456to 0.969 lower) |  | LOW | IMPORTANT |
| **Disability symptom stage subgroup analysis (Non-chronic stage)** | | | | | | | | | | | | |
| 4 | randomised trials | serious^1^ | very serious^2^ | no serious indirectness | no serious imprecision | none | 103 | 105 | Hedges’ g 0.005  lower (-0.749 to 0.759 lower) |  | LOW | IMPORTANT |

^1^ Risk of bias was downgraded because most of the included RCTs were at moderate risk of bias due to incomplete data.

^2^ There were heterogeneity I^2^ value >50%

CI, confidence interval; MD, mean difference;

**Table S5 Data handling and outcomes**

| **First author, year** | **Outcome measurement** | **Data extraction/management of pain intensity** | **Data extraction/management of disability** |
| --- | --- | --- | --- |
| Ahmed, 2020 | Pain: VAS  Disability: NDI | Pre-mean and pre-SD were directly extracted from Table 1.  Post-mean and post-SD were directly extracted from Table 2.  Pooling mean and SD using formula on the laser group and the control group. | Pre-mean and pre-SD were directly extracted from Table 1.  Post-mean and post-SD were directly extracted from Table 2.  Pooling mean and SD used formula on laser group and control group. |
| Alghadir, 2020 | Pain: VAS | Pre-mean and pre-SD were directly extracted from Table 1.  Post-mean and post-SD were directly extracted from Table 1.  Pooling mean and SD used formula on Group A and Group B. |  |
| Buttagat, 2021 | Pain: VAS  Disability: NDI | Pre-mean and pre-SD were directly extracted from Table 2.  Post-mean and post-SD were directly extracted from Table 2.  Pooling mean and SD used formula on Thai massage group and control group. | Pre-mean and pre-SD were directly extracted from Table 2.  Post-mean and post-SD directly extracted from Table 2.  Pooling mean and SD used formula on Thai massage group and control group. |
| El Laithy, 2018 | Disability: NPAD |  | Pre-mean and pre-SD were directly extracted from Table3 and Table 4.  Post-mean and post-SD were directly extracted from Table 3 and Table 4. |
| Joshi, 2022 | Pain: NPRS  Disability: NDI | Pre-mean and pre-SD were directly extracted from Table 2.  Post-mean and post-SD were directly extracted from Table 2. | Pre-mean and pre-SD were directly extracted from Table 2.  Post-mean and post-SD were directly extracted from Table 2. |
| Junaid, 2020 | Pain: NPRS  Disability: NDI | Pre-median and pre-IQR were directly extracted from Table 3.  Post-median and post-IQR were directly extracted from Table 3.  Median ≈Mean.  IQR to SD use formula.  Pooling mean and SD used formula on the myofascial trigger point release and routine physical therapy group. | Pre-median and pre-IQR were directly extracted from Table 3.  Post-median and post-IQR were directly extracted from Table 3.  Median ≈Mean.  IQR to SD using formula.  Pooling mean and SD using formula on the myofascial trigger point release and routine physical therapy group. |
| Kashyap, 2018 | Pain: VAS  Disability: NDI | Pre-mean and pre-SD were directly extracted from Table 2.  Post-mean and post-SD were directly extracted from Table 2.  Pooling mean and SD used formula on Group A and Group C. | Pre-mean and pre-SD were directly extracted from Table 2.  Post-mean and post-SD were directly extracted from Table 2.  Pooling mean and SD used formula on Group A and Group C. |
| Khan, 2022 | Pain: VAS  Disability: NDI | Pre-mean and pre-SD were directly extracted from Table 3.  Post-mean and post-SD were directly extracted from Table 3. | Pre-mean and pre-SD were directly extracted from Table 3.  Post-mean and post-SD directly extracted from Table 3. |
| Kumar, 2015 | Pain: VAS  Disability: NDI | Pre-mean and pre-SD were directly extracted from Table 1.  Post-mean and post-SD were directly extracted from Table 1.  Pooling mean and SD used formula on Group A and Group B. | Pre-mean and pre-SD were directly extracted from Table 3.  Post-mean and post-SD were directly extracted from Table 3.  Pooling mean and SD used formula on Group A and Group B. |
| Kumari, 2016 | Pain: VAS  Disability: NDI | Pre-mean and pre-SD were directly extracted from Table 3.  Post-mean and post-SD were directly extracted from Table 3.  Pooling mean and SD used formula on Group B and Group C. | Pre-mean and pre-SD were directly extracted from Table 6.  Post-mean and post-SD were directly extracted from Table 6.  Pooling mean and SD used formula on Group B and Group C. |
| Lytras, 2020 | Pain: VAS  Disability: NDI | Pre-mean and pre-SD were directly extracted from Table 2.  Post-mean and post-SD were directly extracted from Table 2. | Pre-mean and pre-SD were directly extracted from Table 2.  Post-mean and post-SD were directly extracted from Table 2. |
| Manzoor, 2021 | Pain: VAS  Disability: NDI | Pre-mean and pre-SD were directly extracted from Table 1.  Post-mean and post-SD were directly extracted from Table 1. | Pre-mean and pre-SD were directly extracted from Table 1.  Post-mean and post-SD were directly extracted from Table 1. |
| Nugraha, 2020 | Pain: VAS  Disability: NPNPQ | Pre-mean and pre-SD were directly extracted from Table 3.  Post-mean and post-SD were directly extracted from Table 3. | Pre-mean and pre-SD were directly extracted from Table 3.  Post-mean and post-SD were directly extracted from Table 3. |
| Osama, 2021 | Pain: NPRS | Pre-mean and pre-SD were directly extracted from Table 1.  Post-mean and post-SD were directly extracted from Table 3.  Pooling mean and SD used formula on the autogenic inhibition MET and reciprocal inhibition MET group. |  |
| Phadke, 2016 | Pain: VAS  Disability: NDI | Pre-mean and pre-SD were directly extracted from Table 2.  Post-mean and post-SD were directly extracted from Table 2. | Pre-mean and pre-SD were directly extracted from Table 2.  Post-mean and post-SD directly extracted from Table 2. |
| Revathy, 2016 | Pain: VAS | Pre-mean and pre-SD were directly extracted from Table 3 and Table 4.  Post-mean and post-SD were directly extracted from Table 3 and Table 4. |  |
| Sachdeva, 2019 | Pain: MPQ  Disability: NDI | Pre-mean and pre-SD were directly extracted from Table 5.  Post-mean and post-SD were directly extracted from Table 5. | Pre-mean and pre-SD were directly extracted from Table 8.  Post-mean and post-SD were directly extracted from Table 8. |
| Sadria, 2017 | Pain: VAS | Pre-mean and pre-SD were directly extracted from Table 3.  Post-mean and post-SD were directly extracted from Table 3. |  |
| Sata, 2012 | Pain: VAS  Disability: NDI | Pre-mean and pre-SD were directly extracted from Table 1.5 and Table 1.6.  Post-mean and post-SD were directly extracted from Table 1.5 and Table 1.6. | Pre-mean and pre-SD were directly extracted from Table 1.5 and Table 1.6.  Post-mean and post-SD were directly extracted from Table 1.5 and Table 1.6. |
| Shadmehr, 2022 | Pain: VAS  Disability: NDI | Pre-mean and pre-SD were directly extracted from Table 1.  Post-mean and post-SD were directly extracted from Table 1. | Pre-mean and pre-SD were directly extracted from Table 1.  Post-mean and post-SD were directly extracted from Table 1. |
| Shady, 2021 | Pain: VAS  Disability: NDI | Pre-mean and pre-SD were directly extracted from Table 1 and Table 2.  Post-mean and post-SD were directly extracted from Table 1 and Table 2. | Pre-mean and pre-SD were directly extracted from Table 1 and Table 2.  Post-mean and post-SD were directly extracted from Table 1 and Table 2. |
| Shah, 2015 | Pain: VAS | Pre-mean and pre-SD were directly extracted from Table 2.  Post-mean and post-SD were directly extracted from Table 2. |  |
| Tank, 2018 | Pain: VAS  Disability: NDI | Pre-mean and pre-SD were directly extracted from Table 2.  Post-mean and post-SD were directly extracted from Table 2. | Pre-mean and pre-SD were directly extracted from Table 4.  Post-mean and post-SD were directly extracted from Table 4. |
| Yadav, 2015 | Pain: VAS  Disability: NDI | Pre-mean and pre-SD were directly extracted from Table 3.  Post-mean and post-SD were directly extracted from Table 3.  Pooling mean and SD used formula on Group A and Group B. | Pre-mean and pre-SD were directly extracted from Table 2.  Post-mean and post-SD were directly extracted from Table 2.  Pooling mean and SD used formula on Group A and Group B. |
| Yeganeh Lari, 2016 | Pain: VAS | Pre-mean and pre-SEM were directly extracted from Table 2.  Post-mean and post-SEM were directly extracted from Table 2.  SEM to SD used formula. |  |
| Zibiri, 2019 | Pain: VAS  Disability: NDI | Pre-mean and pre-SD were directly extracted from Table 2.  Post-mean and post-SD were directly extracted from Table 2.  Pooling mean and SD used formula on Group 2 and Group 3. | Pre-mean and pre-SD were directly extracted from Table 2.  Post-mean and post-SD were directly extracted from Table 2.  Pooling mean and SD used formula on Group 2 and Group 3. |

MPQ, Mcgill Pain Questionnaire; NDI, neck disability; NPRS, Numeric Pain Rating Scale; NPAD, Neck Pain and Disability Scale; NPNPQ, Northwick Park Neck Pain Questionnaire; VAS, Visual Analogue Scale; SD: standard deviation.

**Figure S1**. Summary of quality assessment of studies included in the meta-analysis using Cochrane risk of bias 2 tool


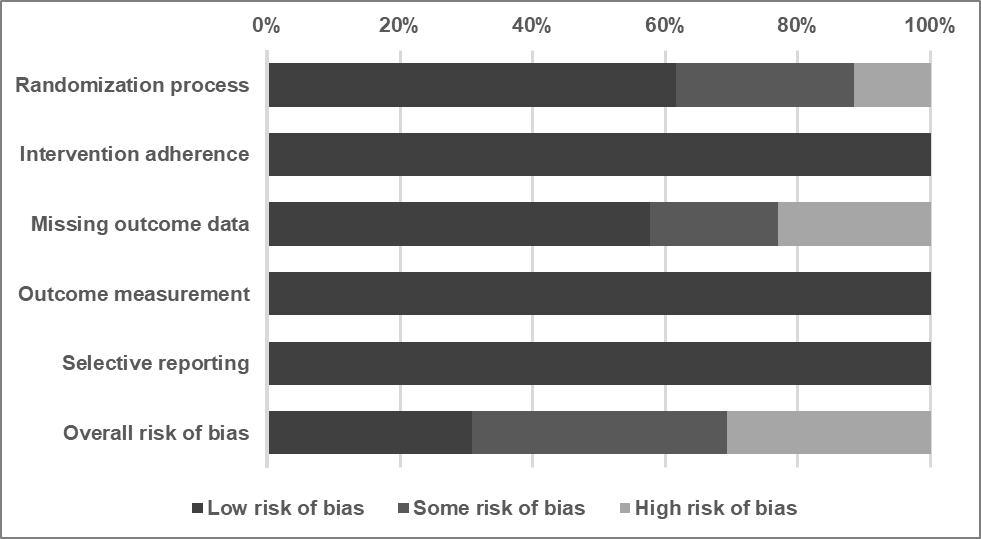


**Figure S2**. Results of sensitivity analysis using the one-study removal method to assess the impact of muscle energy technique (MET) on the overall effect size for pain reduction


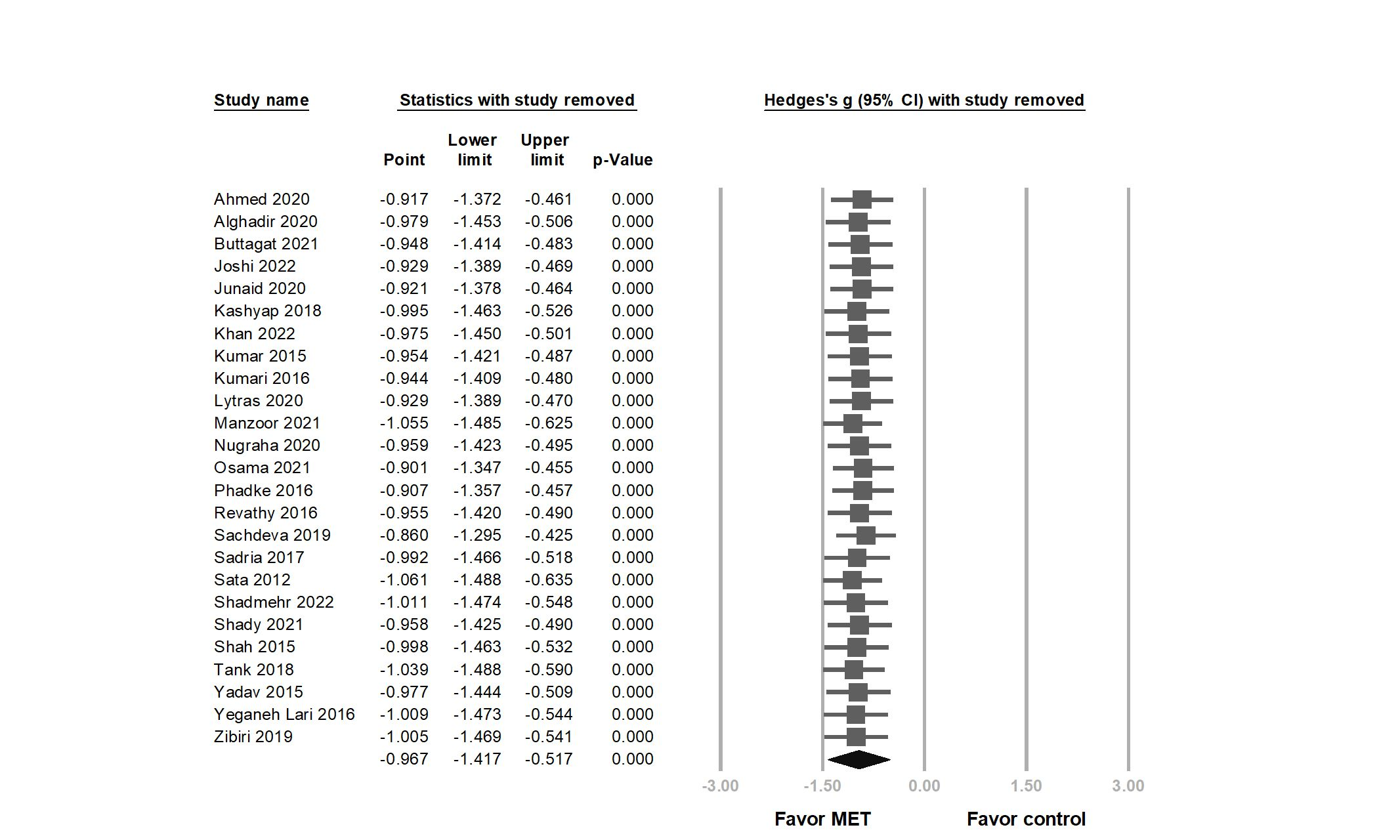


**Figure S3**. Meta-regression analysis showing the relationship between the duration of muscle energy technique (MET) and magnitude of pain reduction


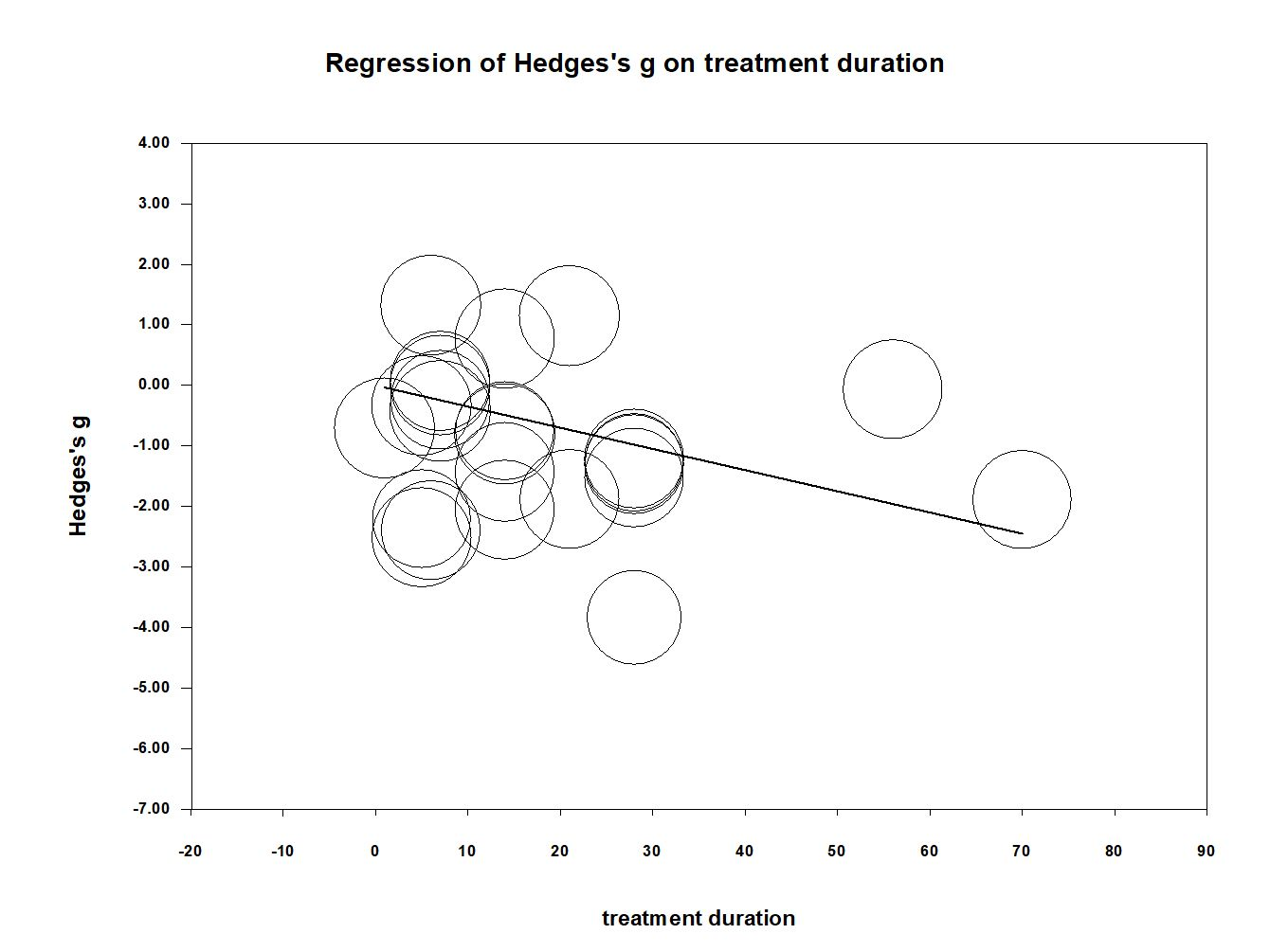


**Figure S4**. Meta-regression analysis showing the relationship between the treatment sessions per week and magnitude of pain reduction


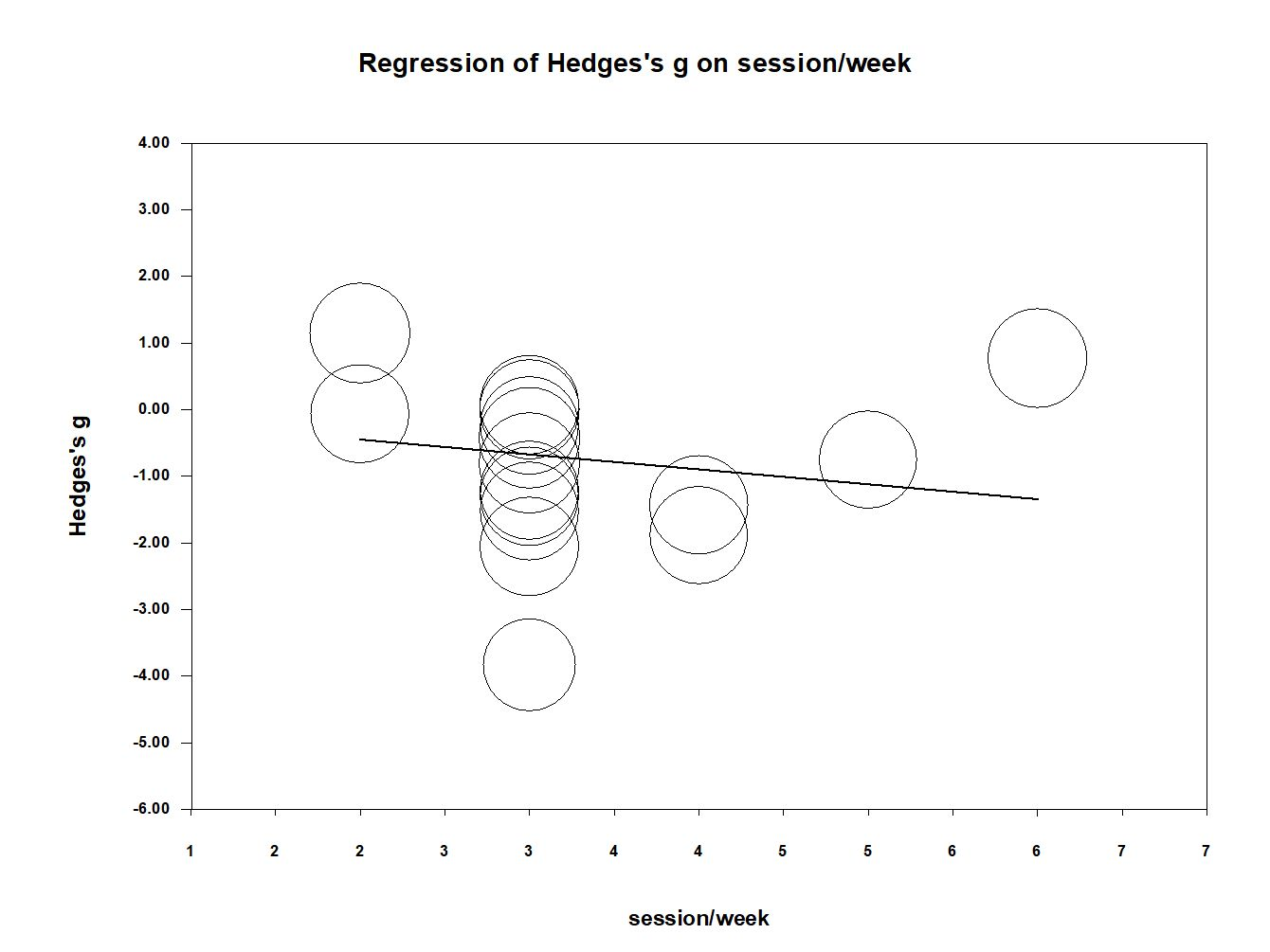


**Figure S5**. Results of sensitivity analysis using the one-study removal method to assess the impact of muscle energy technique (MET) on the

overall effect size for relief of disability


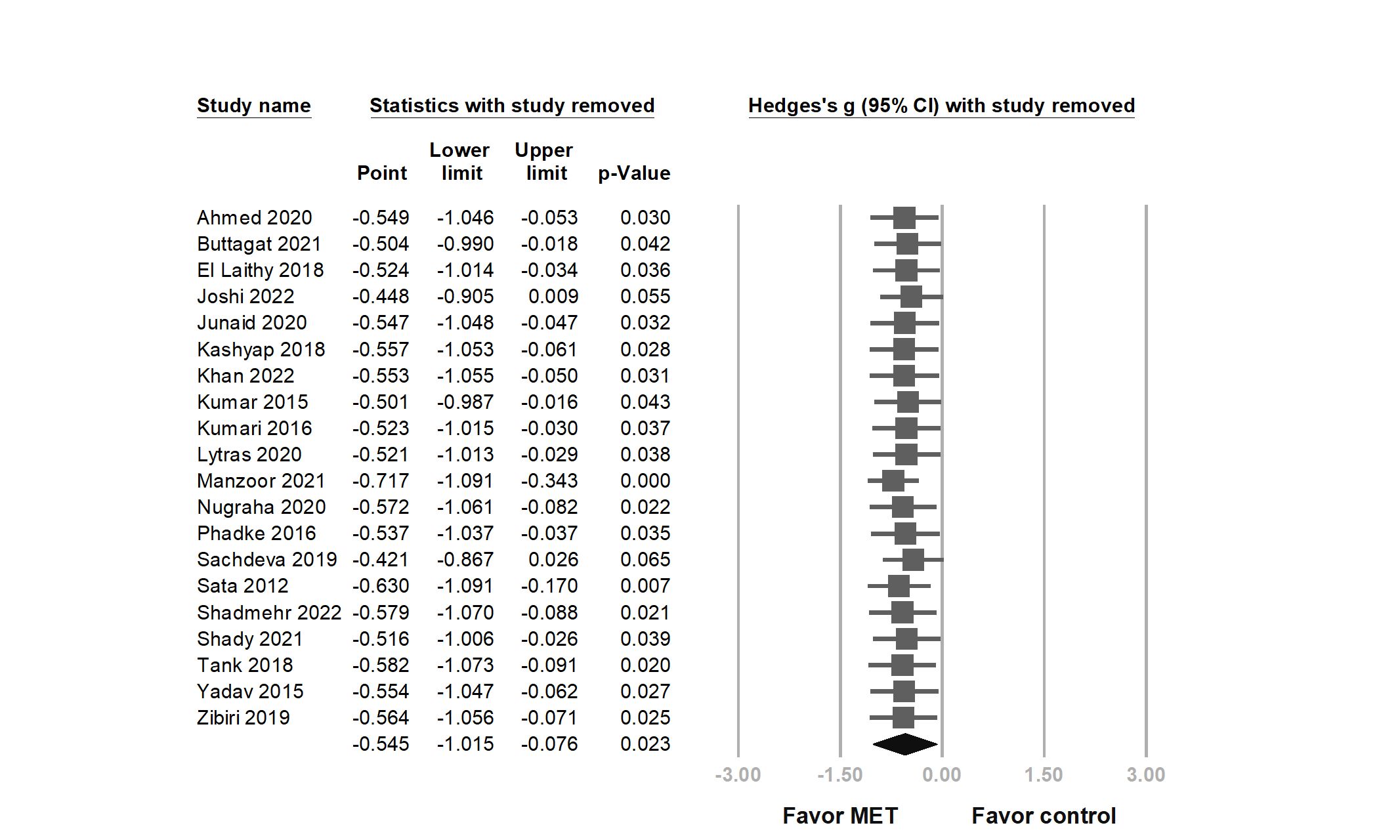


**Figure S6**. Meta-regression analysis showing the relationship between the treatment duration and the magnitude of disability


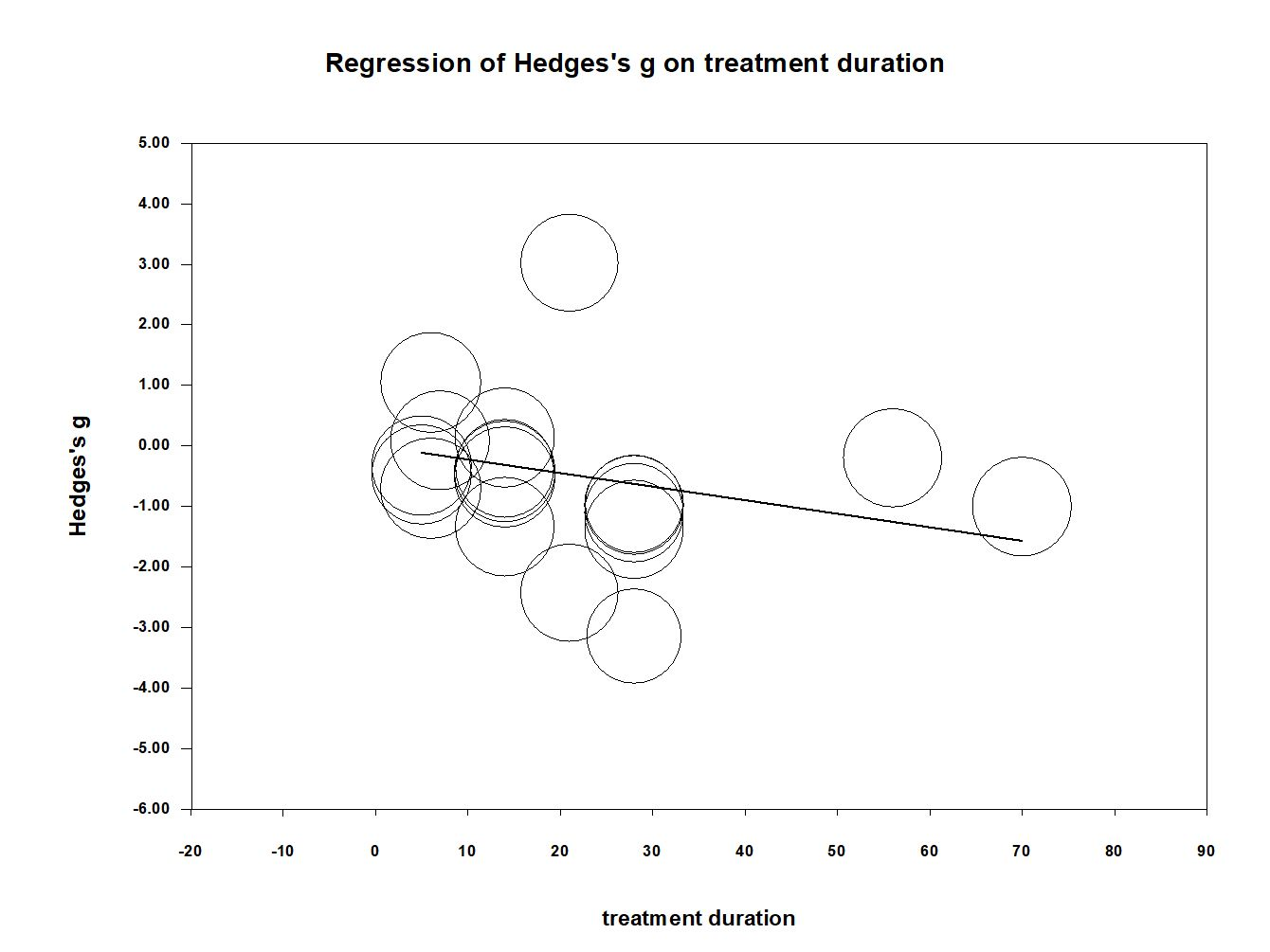


**Figure S4**. Meta-regression analysis showing the relationship between the treatment sessions per week and the magnitude of disability


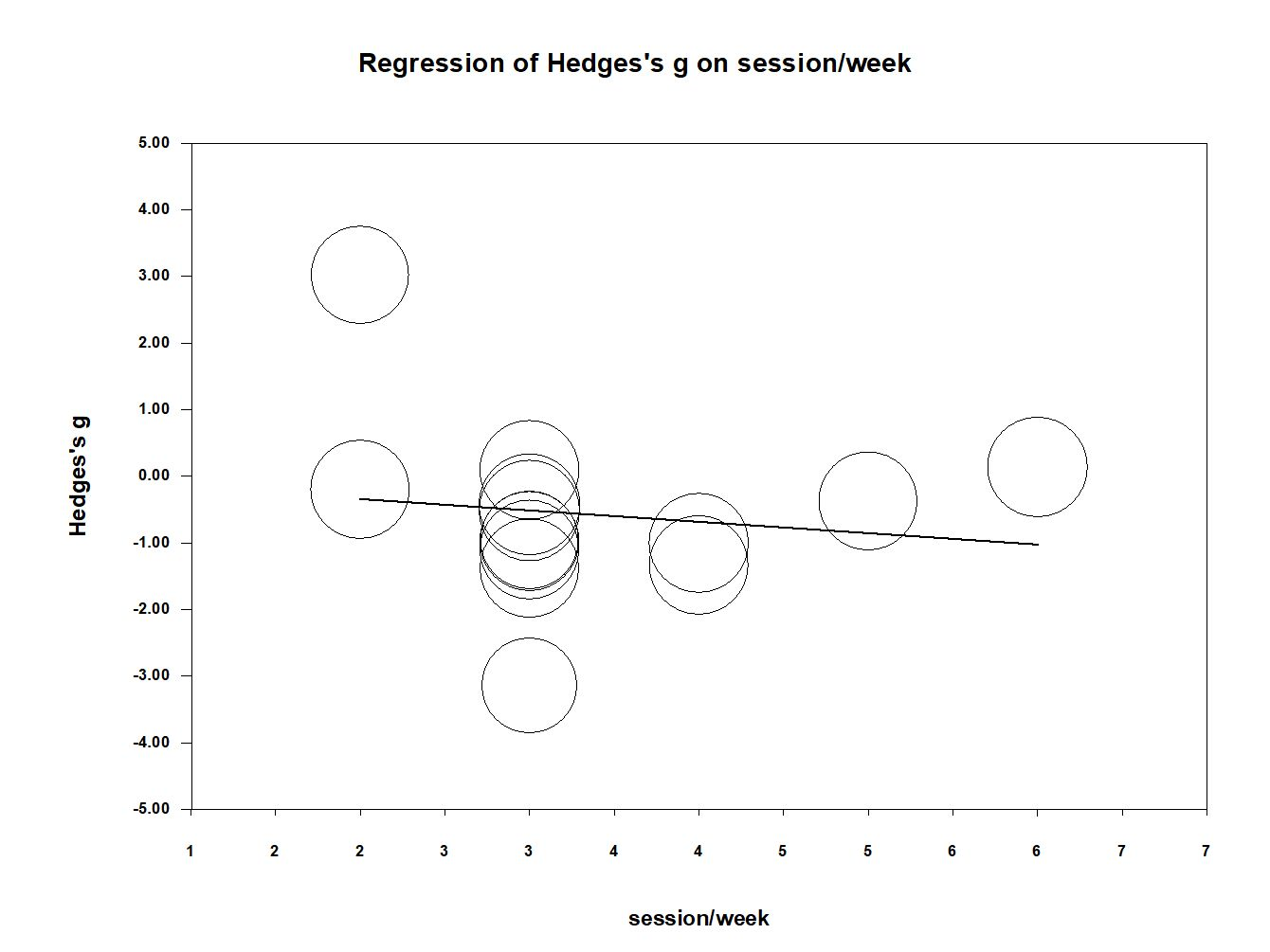


**Figure S8**. Funnel plot depicting the distribution of effect sizes for pain reduction across studies


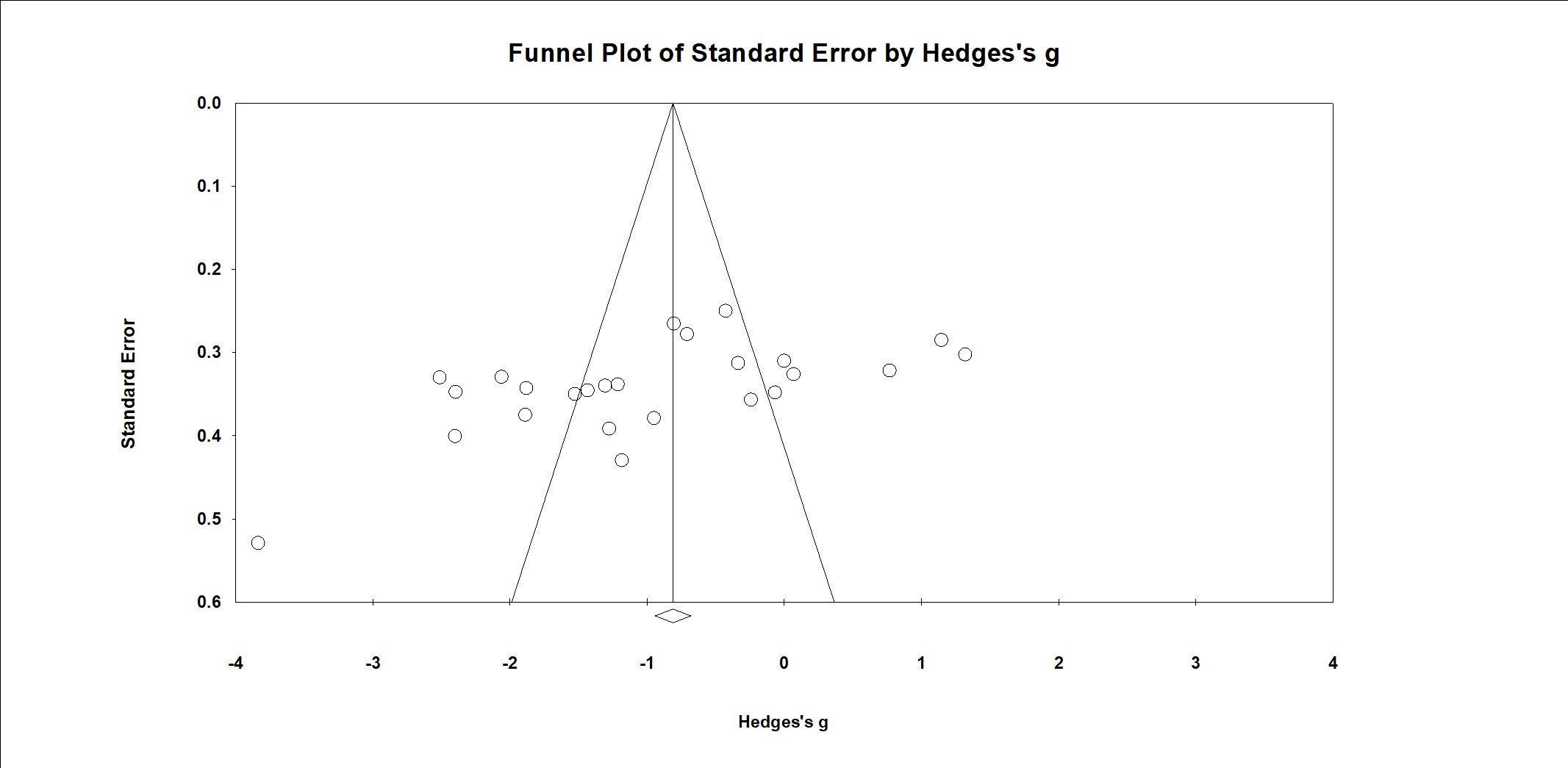


**Figure S9**. Funnel plot depicting the distribution of effect sizes for reduction of disability across studies


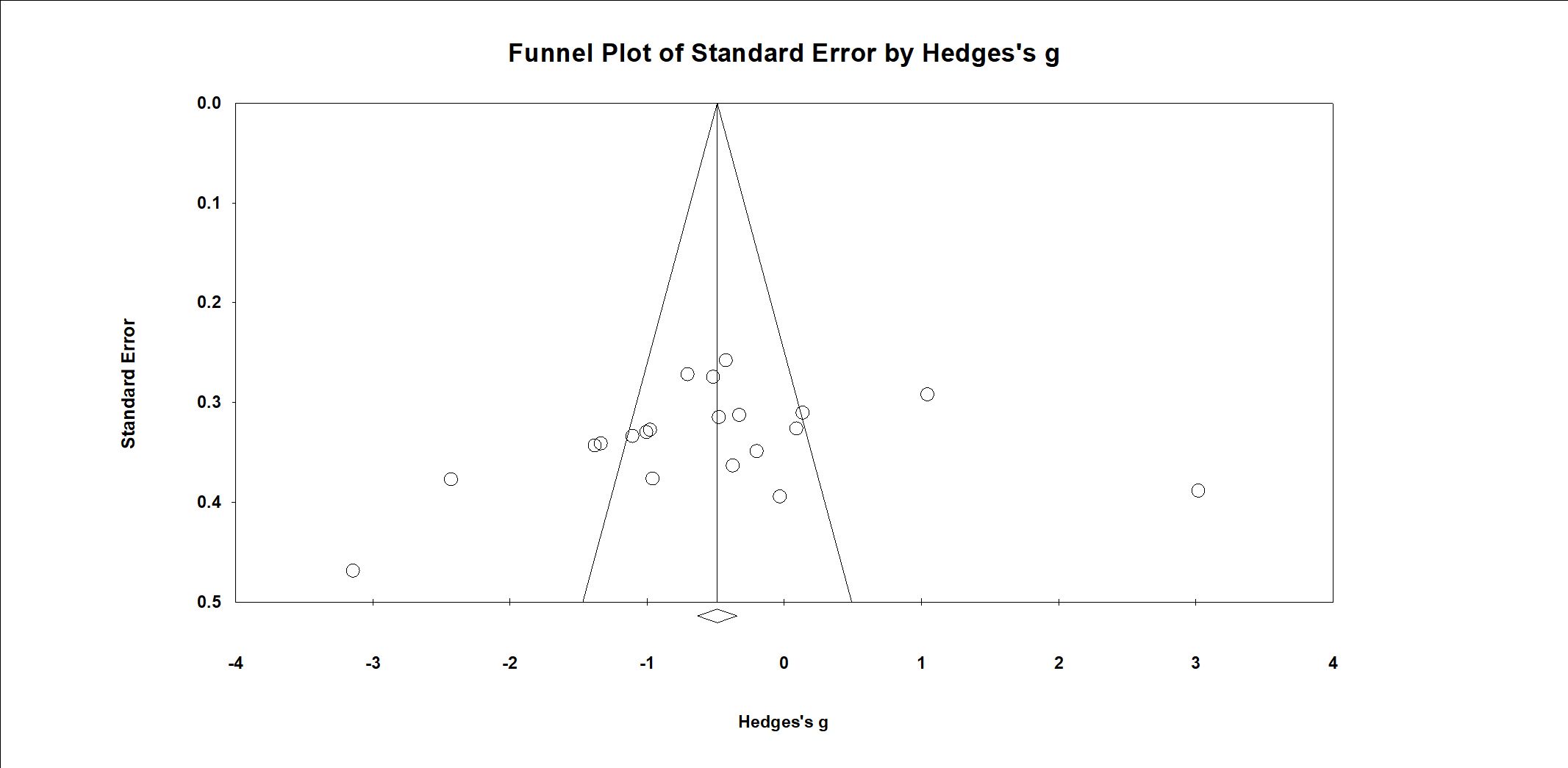

Supplement: Multimedia component 4 [file mmc4.docx]
